# Structural reorganization of the early visual cortex following Braille training in sighted adults

**DOI:** 10.1038/s41598-017-17738-8

**Published:** 2017-12-12

**Authors:** Łukasz Bola, Katarzyna Siuda-Krzywicka, Małgorzata Paplińska, Ewa Sumera, Maria Zimmermann, Katarzyna Jednoróg, Artur Marchewka, Marcin Szwed

**Affiliations:** 1 Department of Psychology, Jagiellonian University, 6 Ingardena Street, 30-060 Krakow, Poland; 2 Laboratory of Brain Imaging, Neurobiology Center, Nencki Institute of Experimental Biology, Polish Academy of Sciences, 3 Pasteura Street, 02-093 Warsaw, Poland; 3 INSERM U 1127, CNRS UMR 7225, Sorbonne Universités, and Université Pierre et Marie Curie-Paris 6, UMR S 1127, Institut du Cerveau et de la Moelle épinière (ICM), F-75013 Paris, France; 4 The Maria Grzegorzewska University, 40 Szczęśliwicka Street, 02-353 Warsaw, Poland; 5 Institute for Blind and Partially-Sighted Children in Krakow,, 6 Tyniecka Street, 30-319 Krakow, Poland; 6 Faculty of Psychology, University of Warsaw, 5/7 Stawki Street, 00-183 Warsaw, Poland; 7 Laboratory of Psychophysiology, Department of Neurophysiology, Nencki Institute of Experimental Biology, Polish Academy of Sciences, 3 Pasteura Street, 02-093 Warsaw, Poland

## Abstract

Training can induce cross-modal plasticity in the human cortex. A well-known example of this phenomenon is the recruitment of visual areas for tactile and auditory processing. It remains unclear to what extent such plasticity is associated with changes in anatomy. Here we enrolled 29 sighted adults into a nine-month tactile Braille-reading training, and used voxel-based morphometry and diffusion tensor imaging to describe the resulting anatomical changes. In addition, we collected resting-state fMRI data to relate these changes to functional connectivity between visual and somatosensory-motor cortices. Following Braille-training, we observed substantial grey and white matter reorganization in the anterior part of early visual cortex (peripheral visual field). Moreover, relative to its posterior, foveal part, the peripheral representation of early visual cortex had stronger functional connections to somatosensory and motor cortices even before the onset of training. Previous studies show that the early visual cortex can be functionally recruited for tactile discrimination, including recognition of Braille characters. Our results demonstrate that reorganization in this region induced by tactile training can also be anatomical. This change most likely reflects a strengthening of existing connectivity between the peripheral visual cortex and somatosensory cortices, which suggests a putative mechanism for cross-modal recruitment of visual areas.

## Introduction

Sensory and motor training induces changes in grey and white matter^1,2^. While a substantial body of research exists on this phenomenon, most of it pertains to local changes within the specific sensory system being trained, such as plasticity of the visual cortex in response to visual or visuo-motor training^3–5^, or plasticity of the auditory cortex in response to audio-motor training^6^. The anatomical mechanisms associated with training that relies on cross-modal plasticity spanning distant brain systems remain largely unknown.

Cross-modal plasticity is known to occur when the brain adapts to changes in the input that it receives, notably changes caused by sensory deprivation^7,8^. In the blind, for example, the visual cortex becomes recruited for auditory and tactile processing, such as Braille reading^9–13^, while in the deaf, the auditory cortex becomes recruited for visual and tactile processing^14–18^. This phenomenon exists also in non-deprived subjects; in professional pianists, the auditory cortex becomes recruited for visuomotor processing^19^; in sighted Braille readers, the ventral visual stream becomes recruited for tactile Braille reading^20^, while in users of Sensory Substitution Devices which transform vision to audition, the ventral visual stream becomes recruited for auditory object perception^21^. Characteristically, such reorganization can also affect low-level sensory cortices^22,23^.

While there are some substantial studies on the anatomical underpinnings of cross-modal plasticity, they nonetheless have two major limitations. First, almost all of them have been performed in sensory-deprived subjects. Second, none of them used a longitudinal design. We do know then that anatomical reorganization occurs in the visual cortex of the blind^24–27^ and the auditory cortex of the deaf^28–32^, and, importantly, several studies show that these changes can be related to cross-modal plasticity rather than to sensory deprivation itself^27,31,32^. However, it is unclear whether similar anatomical mechanisms operate in non-deprived individuals.

We reasoned that a longitudinal study in adult, non-deprived subjects could demonstrate to what degree large-scale anatomical flexibility of the brain is preserved beyond the typical developmental windows characterized in childhood. In addition, we wanted to determine the extent in which anatomical and functional changes are related to each other in the course of cross-modal plasticity. These two facets of the same phenomenon were studied mostly in separate subject samples enrolled in different tasks and trainings. We sought to apply a multimodal imaging approach, which combines structural and functional methods in the same group.

In our recent study, we showed that after nine months of training, tactile Braille reading recruits the ventral visual cortex in sighted adults^20^. Here, we used voxel-based morphometry (VBM) and diffusion tensor imaging (DTI) data from the same group of subjects to describe structural reorganization associated with learning this skill. Additionally, we used resting-state fMRI data to relate potential structural changes to the long-distance functional connectivity between visual and somatosensory-motor cortices. Based on our previous study^20^ and results from blind subjects^27^, we expected to observe anatomical reorganization induced by the Braille training in the visual cortex. Furthermore, since Braille reading is a tactile-motor task, we expected that this anatomical reorganization will occur in a section of the visual cortex that has inherent functional connections to somatosensory and motor cortices.

## Results

Braille training was completed by 29 sighted adults, mostly Braille educators and special education studies students, specializing in blindness and related disabilities (experimental group). Before training, the subjects were familiar with visually-presented Braille but unable to read it tactually (see Behavioural Results; see Supplementary Information online for discussion of potential impact of subjects’ prior familiarity with visual Braille, and of visual Braille learning during the study on our results). The same subject group has been already described in our previous articles^20,33,34^. All subjects from the experimental group underwent baseline and after-training MRI sessions. Additionally, 19 out of 29 originally recruited subjects participated in a follow-up MRI session, nine months after the end of the training. To ensure that observed anatomical changes were specific to the experimental group, we also recruited a longitudinal control group of 15 subjects (see Methods). Subjects in this group were not familiar with Braille reading and received no training. They were scanned at a baseline session and then at the eighteen-month interval, corresponding to the baseline and follow-up sessions in the experimental group. Finally, another group of 31 control subjects was included to test for generality of effects observed in the resting-state fMRI functional connectivity analysis in the experimental group (see Methods).

### Behavioural results

**Figure 1:**
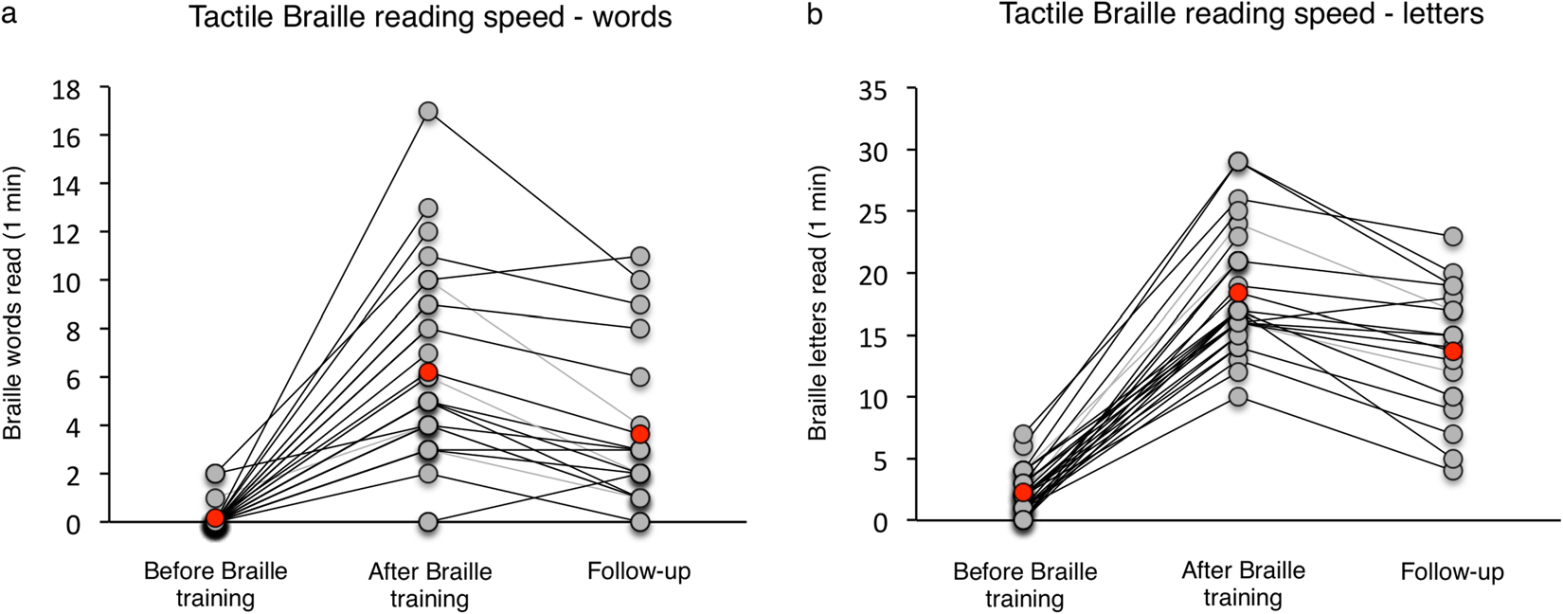
Behavioural outcomes of the tactile Braille training. Individual results of (**a**) the Braille word reading test and (**b**) the Braille letter reading test were plotted for baseline, after-training and follow-up sessions. Group means are illustrated as red dots.

Detailed behavioural results are reported elsewhere^33,34^. Briefly, at the beginning of the study, subjects from the experimental group were familiar with visually-presented Braille but unable to read it tactually. At the baseline testing session, 26 subjects from the experimental group (out of 29 included in this study) were unable to read tactually even a single Braille word per minute (Fig. 1a). One subject managed to read one word and two subjects read two words. Moreover, subjects could recognize tactually only a few Braille letters, and their mean letter reading speed was 2.28 letters per minute (Fig. 1b; LPM; SD = 1.72; range = 0–7). This was in contrast to their reading skills in visually-presented Braille. Even in the baseline session, subjects from the experimental group were able to perform a lexical decision task on Braille words and pseudowords presented visually (see Methods; mean accuracy = 78%, mean reaction time = 4.6 s.; see Supplementary Fig. S1).

During training, subjects from the experimental group significantly progressed in their tactile Braille reading skills. In the after-training testing session, their word reading speed was 6.2 words per minute (Fig. 1a; WPM; SD = 3.94; range = 0–17; after-training vs. baseline session: t(28) = 7.99, p < 0.001) and their letter reading speed was 18.4 LPM (Fig. 1b; SD = 4.81; range = 10–29; after-training vs. baseline session: t(28) = 19.87, p < 0.001). During training, subjects checked their exercises visually (see Methods). Consequently, following training we also observed progress in their visually-presented Braille reading skills. In the lexical decision task, their mean accuracy increased to 88% (Supplementary Fig. S1; after-training vs. baseline session: t(27) = 4.72, p < 0.001) and their mean reaction time decreased to 3.8 s. (Supplementary Fig. S1; after-training vs. baseline session: t(24) = 7.05, p < 0.001)

In the follow-up testing session, the subjects’ tactile Braille reading was slower than in the after-training session – their word reading speed was 3.63 WPM (Fig. 1a; SD = 3.36; range = 0–11; after-training vs. follow-up session t(18) = 4.16, p = 0.001) and their letter reading speed was 13.7 LPM (Fig. 1b; SD = 5.39; range = 4–23; after-training vs. follow-up session t(18) = 5.72, p = 0.001). Nevertheless, 17 out of 19 subjects tested in the follow-up session were still able to read whole Braille words at a speed of at least 1 WPM.

### Neuroimaging results

**Figure 2:**
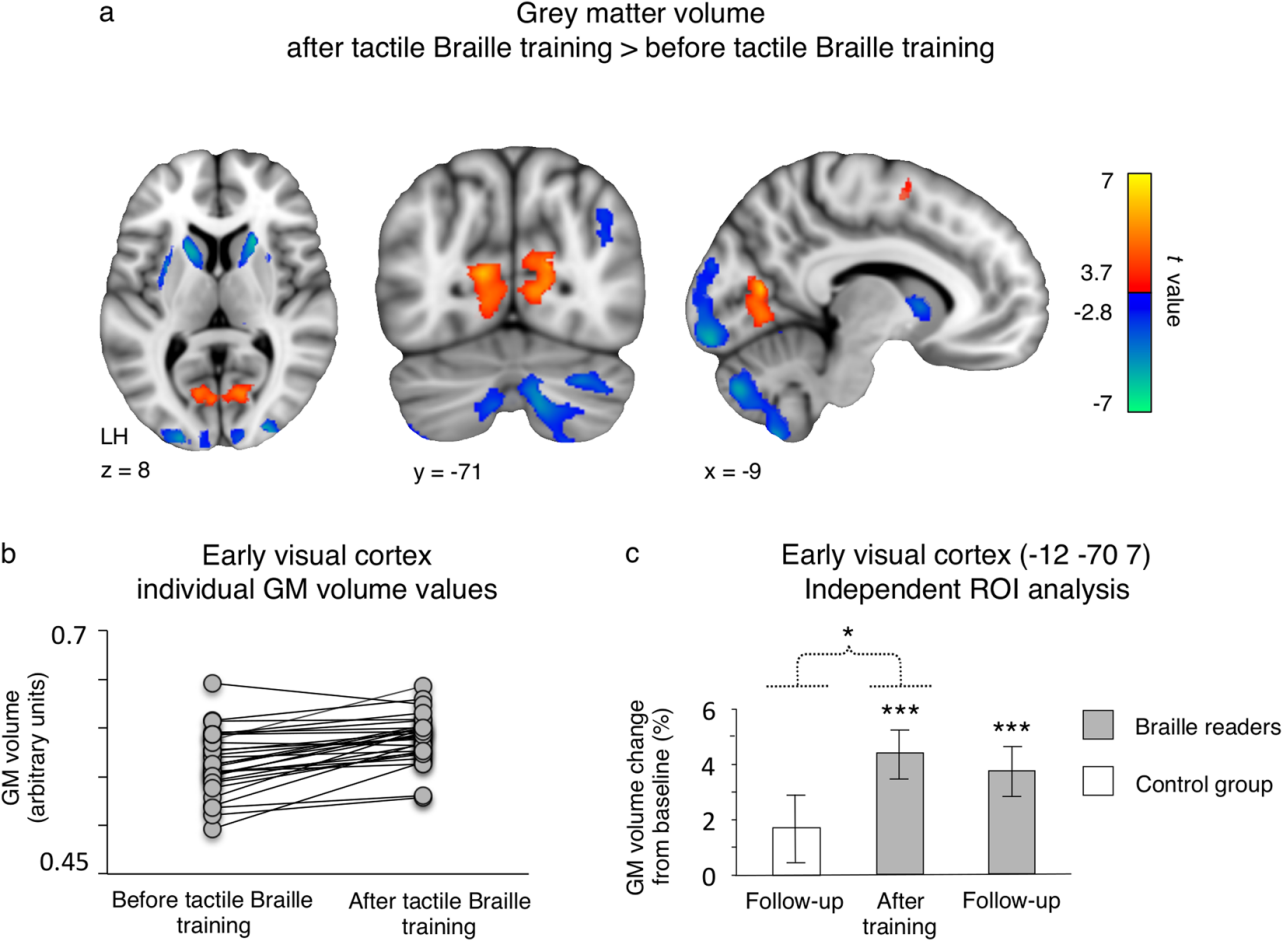
Tactile Braille training induces grey matter volume increases in the peripheral early visual cortex. (**a**) Grey matter (GM) volume changes in the experimental group, following the tactile Braille training. (**b**) Individual subjects’ GM volume changes in the early visual cluster depicted in (**a**). (**c**) The results of ROI analysis in the early visual cortex. Thresholds: (**a**) false discovery rate of q < 0.05, cluster extent threshold of p < 0.05. (**c**) ***p < 0.001; *p < 0.05. The dotted line denotes a time x group interaction. Error bars represent standard error of the mean.

To identify regions in which grey matter volume had been altered following the training, we performed a whole-brain comparison between after-training and baseline scans in the experimental group. Following the training, the most pronounced increase in grey matter volume was observed in the early visual cortex (Fig. 2a; peak MNI = −11–69 12, t = 6.56). This effect was confined to the anterior end of the calcarine sulcus, where the peripheral visual field is represented^35^, and was observed in 27 out of 29 subjects (Fig. 2b). Grey matter volume increases were also found in the left cerebellum and the left premotor cortex (Fig. 2a and Supplementary Table S1). In contrast, we did not observe any significant grey matter volume increases in the control group.

Based on our apriori hypothesis to find anatomical reorganization in the visual cortex, we then applied an independent region-of-interest (ROI) analysis, to describe the time-course of grey matter volume changes in the peripheral early visual cortex and to statistically test whether this reorganization was specific to the experimental group (Fig. 2c; see Methods for ROI definition procedures). This analysis confirmed that in the experimental group, the grey matter volume in the peripheral early visual cortex had increased (after-training vs. baseline scan: t(18) = 4.42, p < 0.001). Moreover, this effect was specific to the experimental group (group x scan interaction: F(1, 29) = 4.66, p = 0.039) and was still present nine months after the end of training (follow-up vs. baseline scan in the experimental group: t(18) = 3.94, p < 0.001; the results of whole-brain follow-up vs. baseline and follow-up vs. after-training VBM analyses can be found in Supplementary Tables S2 and S3). The ROI analysis thus confirmed that tactile Braille training induced grey matter reorganization in the peripheral part of the early visual cortex.

In both groups, we also observed significant grey matter volume decreases with time (baseline vs. after-training contrast in the experimental group and baseline vs. follow-up contrast in the control group; see Fig. 2a and Supplementary Table S1). However, a between-group interaction analysis did not reveal any effects specific to the experimental or the control group.

**Figure 3:**
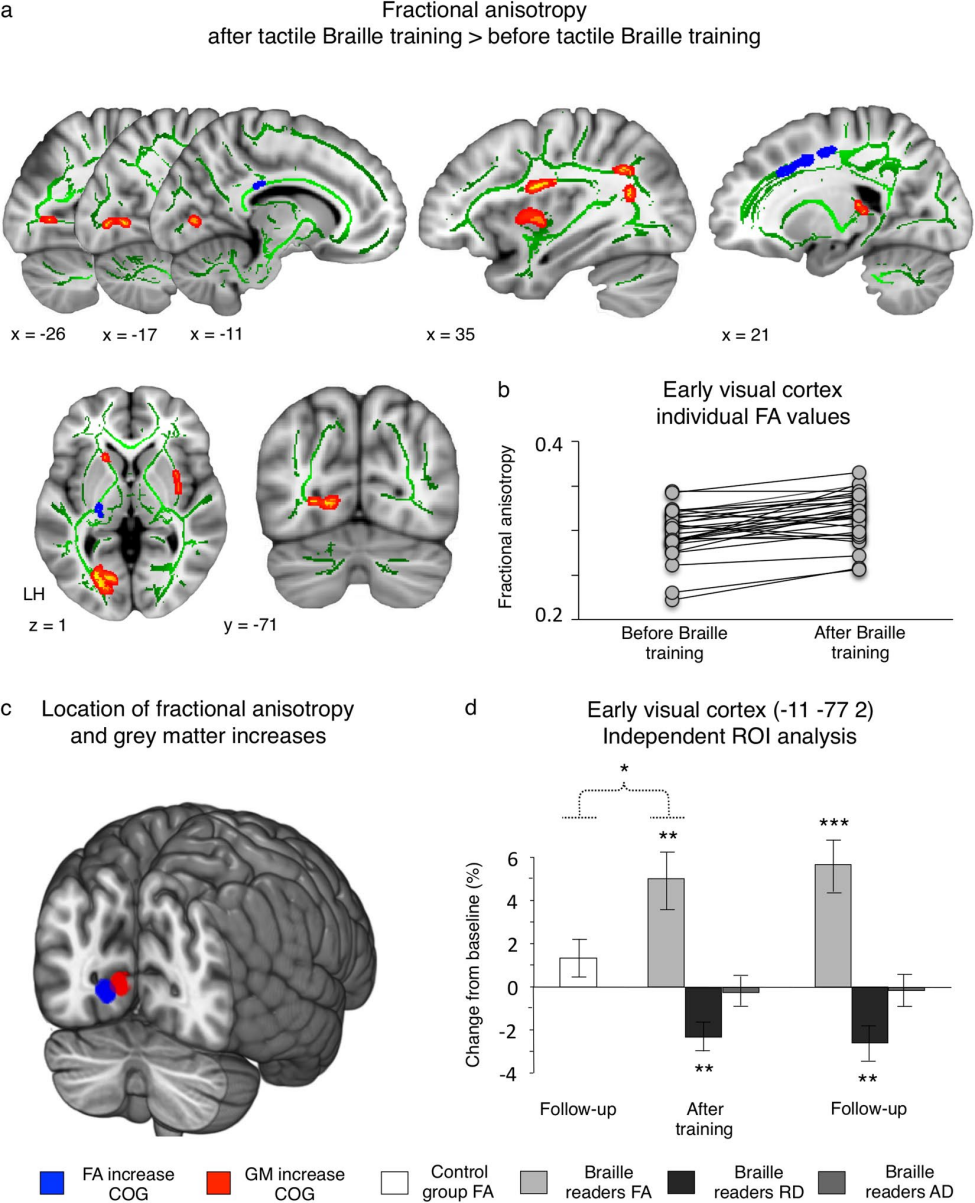
Tactile Braille training induces white matter reorganization in the peripheral early visual cortex. (**a**) Fractional anisotropy (FA) changes in the experimental group, following the tactile Braille training. (**b**) Individual subjects’ FA changes in the early visual cluster depicted in (**a**). (**c**) Centres of gravity (COG) of white matter and grey matter reorganization in the early visual cortex. White matter and grey matter changes are depicted in blue and red respectively. The COGs are illustrated as 6 mm spheres. (**d**) The results of ROI analysis in the early visual cortex. Thresholds: (**a**) p < 0.001, corrected for multiple comparisons using a cluster extent of p < 0.05. Results have been thickened for visualization purposes using the standard *tbss_fill* FSL command, and overlaid on the subjects’ mean FA skeleton (**d**) ***p < 0.001; **p < 0.01; *p < 0.05. The dotted line denotes a time x group interaction. Error bars represent standard error of the mean.

We then performed a whole-brain DTI white-matter connectivity analysis (Fig. 3). Its results were congruent with the pattern of grey matter volume changes. Following tactile Braille training, we observed an increase in fractional anisotropy (FA) in white-matter bundles within the peripheral part of the left early visual cortex (Fig. 3a; peak MNI = −12–75 1, t = 5.04). This increase was observed in 23 out of 29 subjects (Fig. 3b). The location of this effect closely matched the changes in grey matter volume detected with VBM (Fig. 3c). We also found FA increases in frontal lobes and in right-hemipsheric perisylvian regions (see Fig. 3a and Supplementary Table S4). No significant FA increases were observed in the control group.

Next, an independent ROI analysis was applied to the peripheral early visual cortex, using procedures similar to those previously applied to VBM data (Fig. 3d; Methods). This analysis revealed that FA in the peripheral early visual cortex had significantly increased in the experimental group (after-training vs. baseline scan: t(18) = 3.60, p = 0.002). This effect was specific to this group only (group x scan interaction: F(1, 29) = 4.95, p = 0.034) and was still present at nine months after the end of the Braille training (follow-up vs. baseline scan in the experimental group: t(18) = 5.03, p < 0.001; the results of whole-brain follow-up vs. baseline and follow-up vs. after-training DTI analyses can be found in Supplementary Tables S5 and S6). Thus, the ROI analysis confirmed that Braille training induced white-matter reorganization in the peripheral early visual cortex. To facilitate the interpretation of this effect, we also tested for changes in axial diffusivity (AD) and radial diffusivity (RD) in the experimental group (Fig. 3d; Methods). Following Braille training, we observed decreased RD in the peripheral early visual cortex (after-training vs. baseline scan: t(18) = 3.18, p = 0.005). This effect was present also in the follow-up scan (follow-up vs. baseline scan: t(18) = 3.11, p = 0.006). In contrast, no changes were found in AD.

In the experimental group, we also observed significant FA decreases following the Braille training (baseline vs. after-training contrast; Fig. 3a; Supplementary Table S4). While these effects were not detected in the control group (baseline vs. follow-up contrast), no statistically significant between-group interaction was found.

To relate anatomical reorganization in the experimental group to training outcomes, we correlated training-related (after-training – baseline) changes in grey matter volume (VBM analysis) and FA (DTI analysis) with training-related changes in behavioural variables (tactile Braille word reading speed, tactile Braille letter reading speed, visual Braille word reading speed and visual Braille word reading accuracy; see Behavioural Result and Methods) and with age. However, we did not detect any significant correlation, neither in the whole-brain analysis, nor in the ROI analysis of the peripheral early visual cortex (see Supplementary Figure S2). Additionally, the ROI approach was used to correlate training-related changes in grey matter volume and FA in the peripheral early visual cortex with each other. These two variables were positively correlated (R(27) = 0.618, p < 0.001; Supplementary Figure S2).

**Figure 4:**
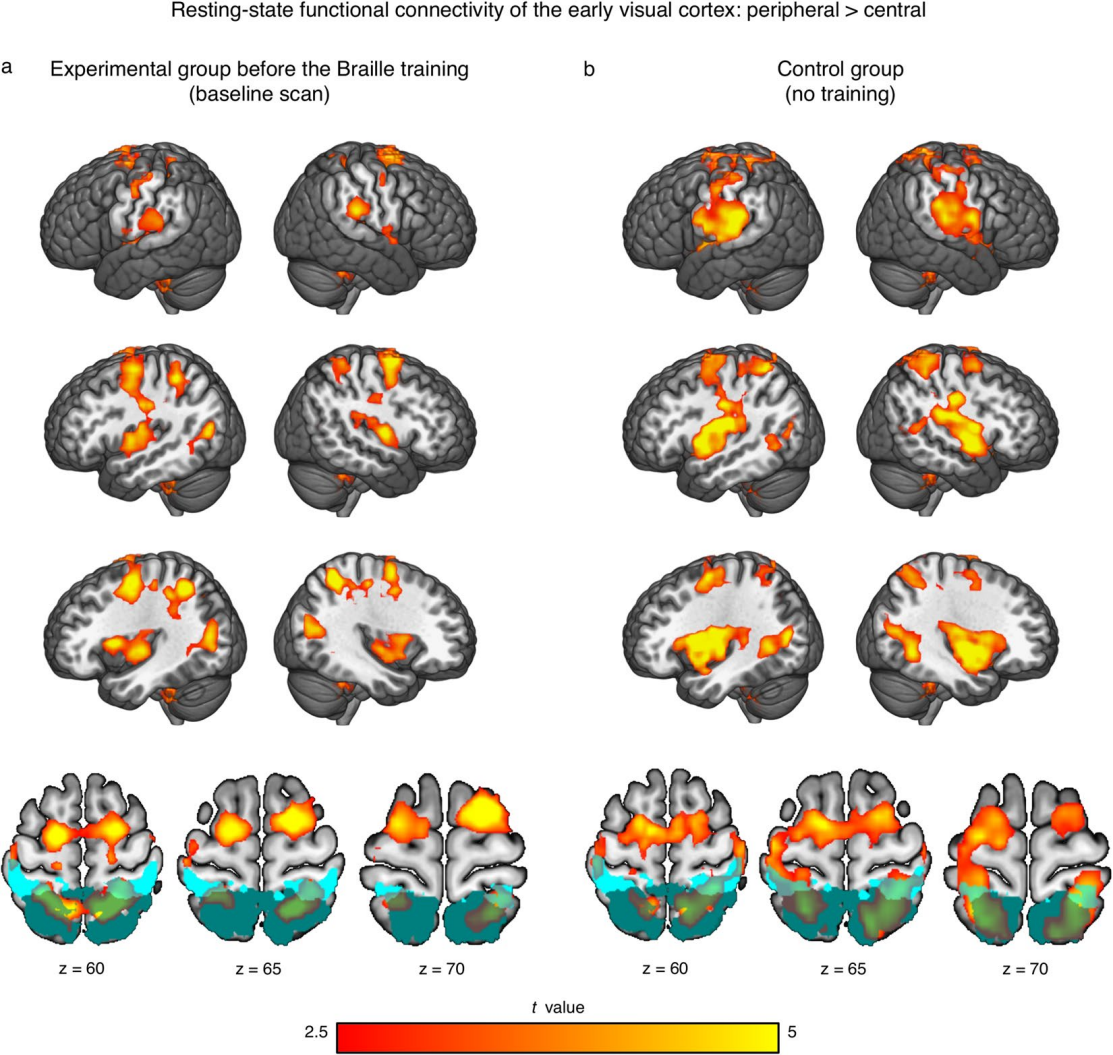
The peripheral part of early visual cortex was preferentially connected to somatosensory and motor cortices even before the onset of Braille training. Before-training resting-state functional connectivity of the anterior part of the early visual cortex (known to represent the peripheral visual field) relative to the posterior part of this region (central visual field). Results are shown for the experimental (**a**) and control (**b**) groups. Thresholds: false discovery rate of q < 0.05, cluster extent threshold of p < 0.05. The primary somatosensory cortex (areas 1, 2, 3a and 3b) is marked with light cyan. The secondary somatosensory cortex (areas 5 and 7) is marked with dark cyan. Masks of somatosensory areas were obtained from the Anatomy SPM toolbox.

Finally, we used resting-state fMRI functional connectivity analysis to test whether the peripheral part of the early visual cortex is preferentially connected to somatosensory-motor cortices. To this aim, we compared, in the experimental group, the before-training functional connectivity of the peripheral early visual cortex with the before-training functional connectivity of the central early visual cortex (seed-to-whole-brain functional connectivity analysis; see Methods). We found that, compared to its central counterpart, the peripheral early visual cortex is preferentially connected with numerous brain regions, including the insula, the inferior frontal gyrus, the parietal lobe and the cerebellum (Fig. 4a; see also Supplementary Table S7). Of particular importance to our hypotheses, we also observed preferential connectivity between the peripheral early visual cortex and somatosensory-motor cortices (Fig. 4a; Supplementary Table S7). Notably, this preferential connectivity was present already before the onset of the Braille training. We did not observe a change in the functional connectivity of peripheral early visual cortex following the training.

To test for generality of effects observed in the resting-state fMRI analysis in the experimental group, the same comparison between functional connectivity of the peripheral early visual cortex and functional connectivity of the central early visual cortex was performed in control subjects (see Methods). Using this independent dataset, we replicated results observed in the experimental group, particularly the preferential functional connectivity between the peripheral early visual cortex and somatosensory-motor cortices (Fig. 4b; Supplementary Table S8).

## Discussion

To the best of our knowledge, this study is the first to describe the anatomical mechanisms associated with learning a skill that relies on cross-modal recruitment of a sensory cortex. An increasing number of studies has been showing that convergence of information from different senses can occur not only in multisensory areas in temporal and parietal lobes^36,37^, but at the level of primary sensory cortices as well^38,39^. Such multisensory effects are particularly well-documented in the early visual cortex^40^, and, in line with these results, non-human anatomical data indicates that early visual areas are monosynaptically connected with distant, non-visual regions, including auditory and somatosensory cortices^41–43^. This long-range connectivity was shown to be denser in the anterior part of the early visual cortex^41,42^, which represents the peripheral visual field^35^.

In our study, it is exactly the peripheral early visual cortex that underwent structural reorganization specifically linked to the tactile Braille reading training (see interaction analyses in Figs 2c and 3d). The effect was observed for both grey and white matter. In the case of white matter, changes were observed mainly in radial diffusivity, which might suggest increased myelination^44–46^ (see Supplementary Information for further discussion). In addition, in line with earlier reports, resting-state fMRI showed that the peripheral early visual cortex is preferentially connected to somatosensory and motor cortices even before the onset of Braille training (see also ref.^47^). This initial “connectivity bias” might have thus underlain the reorganization of this region described in our article (see also^7,48^).

Previous notable studies have already reported functional recruitment of the early visual cortex for non-visual tasks. Following blindness, this region becomes critical for Braille reading, language and memory^49–52^. In the sighted, studies report activations in the early visual cortex induced by tactile and auditory discrimination tasks^23,53–59^. Following several days of blindfolding and tactile training, this region becomes necessary for recognition of single Braille characters^22^. Based on such reports, it was proposed that the early visual cortex can subserve spatial processing, regardless of the modality of input^60^. Intensive training or sensory deprivation might lead to reweighting of visual and non-visual inputs that reach this region, which results in “unmasking” of non-visual activations.

Our data are consistent with this hypothesis. Both a “connectivity bias” and the functional role of early visual cortex in spatial processing of Braille characters might have contributed to the structural plasticity observed in our study. As Reich and colleagues^13^ have speculated, early visual areas might become involved in analysing simple geometrical features of individual Braille letters. In line with this interpretation, in our previous study on Braille reading in sighted subjects, the activation of the early visual cortex was modulated by single Braille letter recognition speed, but not by whole-word Braille reading speed. Whole-word Braille reading speed, in turn, modulated activity in higher-level ventral visual regions^20^.

In our study, we also detected grey matter volume and FA decreases with time (see Figs 2a and 3a as well as Supplementary Tables S1 and S4). However, no interaction between the experimental group and the control group was observed, which suggests that most of observed decreases are likely to be associated with non-specific, physiological effects, such as ageing, which can be detected at timescales similar to the timescale of our study^61–64^ (see also Supplementary Discussion).

In our previous study, we demonstrated that the ventral visual cortex becomes critical for Braille reading in sighted adults^20^. Here, we analysed the same group of subjects and showed that learning to read Braille induces specific, anatomical reorganization in the peripheral early visual cortex. Based on our results, we hypothesize that this change reflects strengthened anatomical connectivity between visual and somatosensory cortices – a mechanism that is likely to support cross-modal recruitment of the visual cortex for tactile input. In combination with previous studies, our data suggests that, as a results of training, tactile input might be first transferred from somatosensory to early visual cortices, most likely for detailed spatial analysis^13,22,55,60^. This information is then further processed in high-level visual areas, which have the capacity to process complex perceptual input and link it with meaning^13,65–67^. Future studies should use methods sensitive to the temporal dynamics of brain computations, such as EEG or chronometric TMS, to verify and further refine the model of training-induced cross-modal plasticity of visual cortices proposed here.

An alternative explanation of our results is that structural reorganization of the early visual cortex reflects visual imagery and is not directly linked to tactile input transfer and processing. In this view, structural changes in the early visual cortex would be triggered by increased reliance on visual images of Braille when one learns to read this script tactually. While we cannot entirely exclude such an explanation, it is highly unlikely that visual imagery alone could account for structural changes observed in the early visual cortex. Our data show that reorganization specific to the Braille training was observed only in the anterior part of this region, which represents the peripheral visual field^35^. Yet, when read visually, Braille characters are viewed in the central visual field, like any other script. If visual imagery was behind the changes in the early visual cortex during the training, one should expect training-related effects centred on the representation of the central visual field. The spatial location of the observed structural changes thus argues against the critical role of visual imagery. Moreover, in our previous fMRI study on the same group of subjects, we showed that visual imagery itself cannot explain visual cortex activations during Braille reading^20^. Thus, converging evidence from functional and structural data suggests that changes in the visual cortex during Braille training are indeed linked to tactile input transfer and processing.

It is interesting that Braille training induced anatomical reorganization in the peripheral early visual cortex without inducing detectable changes in functional connectivity of the same region. In our study, functional connectivity was calculated based on resting-state fMRI data, which is likely to make this measure sensitive to relatively stable and unspecific features of functional networks, persisting even when no task is performed. In contrast, our interpretation of results observed in this study assumes that detected anatomical changes support highly specific functional interactions between the early visual cortex and the somatosensory cortex, which occurs only during complex tactile activities (see above). Such changes might not have direct consequences to resting-state functional connectivity patterns. Notably, in our previous study, we observed that Braille training did induce a change in functional connectivity of the ventral visual cortex^20^, which, as a relatively high-level cortical region, might have higher functional flexibility than the early visual cortex. It is possible, that with time and additional training, also the early visual cortex would change its pattern of resting-state functional connections.

We also did not find correlations between training-induced anatomical reorganization and the behavioural outcomes of the training. However, other studies^5,68,69^ also often fail to find such correlations, which suggests that the relationship between anatomical plasticity and behaviour might be more complex and perhaps mediated by other variables (see also: ref.^70^). It might be the case that anatomical brain reorganization emerges from the interaction between multiple factors, such as the amount of training, its subjective hardness, objective behavioural outcomes and individual neuroplastic capabilities of the cortex.

There are several ways in which the transfer of tactile input from the somatosensory cortex to the peripheral early visual cortex could occur. It could be thus achieved through polysynaptic pathways, leading to the visual system via multisensory regions in parietal lobes^36,42^. Alternatively, tactile-induced recruitment of the visual cortex could be supported by direct connectivity between somatosensory and early visual cortices. A strong and left-lateralized reorganization of white matter in the peripheral early visual cortex, observed in our study (see Fig. 3), seems to support the latter. Indeed, white-matter pathways that directly link the primary somatosensory cortex and the early visual cortex were detected in rodents^43,71^. Nevertheless, the existence of such connectivity in primates remains speculative^72^. In monkeys, one study reported direct connectivity between the peripheral early visual cortex and area PE, which is considered to be analogous to the human secondary somatosensory cortex^42^. However, it remains to be determined whether similar pathways exist in humans, and whether tactile-induced recruitment of the visual cortex is indeed supported by direct, monosynaptic connectivity.

Whichever route, parietal of direct, turns out to be valid, we can nonetheless conclude that training in tactile Braille reading induces anatomical reorganization in the visual cortex. This suggests that the adult human brain can adapt its structural properties to support a global change in the cortical processing hierarchy and that this flexibility (see also ref.^73^) might underlie the distinctively human capacity to efficiently learn complex skills.

## Methods

### Subjects

The experimental group included 29 subjects (26 females, 3 males; mean age = 29.34; SD = 7.52; range = 22–49), mostly Braille educators and special educations studies’ students, specializing in blindness and related disabilities. Before the study, they were familiar with reading visually presented Braille, but unable to read it tactually (see Behavioural Results). All were right-handed, fluent in Polish, had normal or corrected-to-normal vision and no neurological or psychiatric disorders. All subjects underwent an MRI session before the onset of nine-month Braille training (baseline session) and immediately after its end (after-training session). Nineteen subjects participated in a follow-up MRI session nine months after the training had ended. All declared that they had not trained in tactile Braille reading after the training had ended. Data from the same subjects were reported in our previous studies^20,33,34^.

In addition, 15 individuals (12 females, 3 males; mean age = 25.53; SD = 3.30; range = 21–32) participated in the study as a longitudinal control group. All were right-handed, fluent in Polish, had normal or corrected-to-normal vision, no neurological or psychiatric disorders and no familiarity with tactile or visual Braille reading. The control group did not undergo training. It was matched for age, sex and education to the experimental group (all p > 0.05). The size of the control group was similar to the size of the experimental group at the follow-up session, for unbiased ROI interaction analyses (see Statistical Analysis section). The control group was scanned twice: first at a baseline session and then in a follow-up session after 18 months. The control-group scans corresponded with the baseline and follow-up sessions in the experimental group.

To test for generality of effects observed in the resting-state fMRI functional connectivity analysis of the experimental group, we performed an additional resting-state fMRI analysis on a separate control group of 31 subjects (17 females, 14 males; mean age = 27.29; SD = 4.54; range = 19–37; due to time constraints, resting-state fMRI data were not collected from the longitudinal control group). The inclusion criteria were the same as in the longitudinal control group.

The research described in this paper was approved by the Committee for Research Ethics of the Institute of Psychology of the Jagiellonian University (decision 28/06/2012). An informed consent and a consent to publish were obtained from each of the participants. All experiments were performed in accordance with the relevant guidelines and regulations.

### Braille training and behavioural measures

The experimental group underwent Braille training and behavioural testing. Its details are reported elsewhere^33,34^. In short, the Braille training lasted for nine months and was based on individual exercises to be performed while blindfolded. The subjects were instructed to train for approximately 20 minutes each day on one A4 Braille printout. The progress in tactile Braille reading was tested monthly, starting from the 5^th^ month of the course to its end. Additionally, the same tests were performed also in the follow-up session. Subjects were blindfolded and asked to read tactually as many Braille letters as possible in sixty seconds. Then, the same procedure was repeated for whole Braille words.

Subjects from the experimental group were familiar with visually presented Braille at the beginning of the course. During the course, they checked their tactile Braille exercises visually, which constituted a form of visual Braille reading training. To control for potential improvements in visual Braille reading, we measured its speed and accuracy with a lexical decision task (detailed results in ref.^34^). Subjects completed the lexical decision task on visual Braille at the beginning and at the end (i.e., in the 9^th^ month) of the course. They were instructed to visually read Braille strings appearing on the screen for 8 seconds and decide, as fast and as accurately as possible, whether they formed a valid Polish word or not and to indicate their choice with a mouse button.

### MRI and fMRI data acquisition

All data were acquired on the same Siemens MAGNETOM Tim Trio 3 T scanner with a 32-channel head coil. High-resolution T1-weighted images were acquired using a MPRAGE sequence (176 slices; FOV = 256; TR = 2530 ms; TE = 3.32 ms; voxel size = 1 × 1 × 1 mm). Two sets of diffusion-weighted images (64 directions; b-value = 1000 s/mm^2^; FOV = 256; TR = 8700 ms, TE = 92 ms; voxel size = 2 × 2 × 2 mm), along with two images with no diffusion gradient applied (b-value = 0), were also acquired. In addition to structural scans, resting-state fMRI data were collected in the same scanning session. A gradient-echo planar imaging sequence sensitive to blood-oxygen-level dependent contrast (282 volumes; 33 contiguous axial slices; TR = 2190 ms; TE = 30 ms; voxel size = 3 × 3 × 3.6 mm) was used. During the resting-state fMRI scan the subjects were asked to fix their gaze on the point displayed on the screen and to relax, but not fall asleep.

### Data preprocessing

T1-weighted images were preprocessed with VBM8 toolbox (http://dbm.neuro.uni-jena.de/vbm/) and SPM8 software (www.fil.ion.ucl.ac.uk). We used the standard VBM8 longitudinal routine, including: 1) within-subject registration of all scanning time-points 2) intra-subject bias correction 3) segmentation of the different tissues classes 4) linear (i.e., affine) and nonlinear (i.e., DARTEL) registration and normalization to the MNI space. Finally, the grey matter images were smoothed with a 6 mm FWHM Gaussian kernel.

Diffusion-weighted images were preprocessed with the tract-based spatial statistics approach (TBSS)^74^ implemented in the FSL 5.06 software (FMRIB Software Library, FMRIB, Oxford, UK)^75^. TBSS is widely-used and reliable approach to analyse DTI data across the whole-brain^76,77^. Thanks to its automaticity, it overcomes many pitfalls of tractography-based methods (see for example:^78^).

For each subject, diffusion-weighted images were affine registered to the first b0 image to correct for subject motion and eddy current induced distortion. Brain mask was created from the b0 image using BET^79^. Next, the FDT toolbox^80^ was used to fit the tensor model and compute the fractional anisotropy (FA), axial diffusivity (AD) and radial diffusivity (RD) maps^81^. Further processing of the maps was optimized for longitudinal analysis^82,83^. First, the maps were smoothed with a 1-voxel median filter to account for small, within-subject registration errors between time points and increase test-retest reliability. Next, subject-specific templates were created based on data from first two time points (i.e. baseline and after-training in the experimental group, and baseline and follow-up in the control group) For each subject, the maps from these time points were registered to each other and the halfway point was computed between pair of images. The mean images were created by averaging the two registered images for each subject. Then, FA template images were non-linearly transformed on the mean FA template provided by FSL (FMRIB58_FA) and then affine transformed on the standard MNI space. Next, they were used to create the study-specific mean FA image which was skeletonized with a threshold FA > 0.2 to generate the white-matter tract skeleton representing tracts common to all subjects. Individual FA images from both time points were then projected onto the reference skeleton generated in the previous step. Finally, the registration matrices and skeleton projections obtained during processing of FA maps were applied to AD and RD maps. For ROI comparison between the follow-up scan and the baseline scan in the experimental group, these two time points were registered to each other using the same procedure.

The resting-state fMRI data were preprocessed with the DPARSF toolbox (Data Processing Assistant for Resting-State fMRI)^84^ and SPM8 (www.fil.ion.ucl.ac.uk). The first ten volumes of each participant’s scan were discarded for signal stabilization and so that the participants could adapt to the noise of the scanner. Slice-timing correction and head-motion correction were then applied, T1 images were segmented, and both anatomical and functional images were normalized to MNI space. Two steps specific to the functional connectivity analysis - regression of nuisance covariates and bandpass filtering - were performed to reduce spurious variance unlikely to reflect neuronal activity. Nuisance regression was performed first to avoid attenuation of the signal caused by mismatch in the frequencies of the data and regressors^85^. Nuisance variables included: a) white matter signal; b) cerebrospinal fluid signal; c) 24 head motion parameters: 6 parameters of the current volume, 6 of the preceding volume and a quadratic term for each of these values^86^ (see also^87,88^); and d) a separate regressor for every volume that displayed a mean frame displacement value higher than 0.5^89^. Given that global signal regression can disturb meaningful signal^90–92^, we did not include global signal as a regressor. After the regression of nuisance covariates, a bandpass filter (0.01–0.08 Hz) was applied. The resulting images were smoothed with a 5 mm FWHM Gaussian kernel. The functional connectivity measure (FC) was then calculated according to standard procedures. For each seed, subject and scan, we computed a voxel-wise correlation of time courses between seed regions and the rest of the brain. These correlation maps were next transformed into Fisher’s z value maps, which were subsequently used in the statistical analysis.

### Statistical analysis

Similar statistical analyses were applied to VBM and DTI data. In the case of VBM, parametric paired t-tests were applied to compare baseline, after-training and follow-up scans in the experimental group as well as baseline and follow-up scans in the longitudinal control group across the whole-brain. When necessary, the whole-brain interactions between groups were tested in 2 × 2 ANOVAs, with scanning session as a within-subject factor and group as a between-subject factor. These comparisons were assigned a false discovery rate threshold of q < 0.05^93^ and an additional cluster-extent threshold of p < 0.05. In the case of DTI, the same comparisons were performed with permutation (i.e., non-parametric) paired t-tests, implemented in the FSL randomise utility^94^. Given that permutation tests were shown to be more robust than their parametric counterparts^95^, and that only small subset of voxels were included in the DTI comparisons (i.e., only voxels in which FA > 0.2 – see Data Preprocessing), a more lenient voxel-wise threshold was assigned, namely p < 0.001. Results that survived this voxel-wise threshold were then corrected for multiple comparisons using a cluster extent threshold of p < 0.05. In addition, for illustrative purposes, we plotted individual grey matter volume (Fig. 2b) and FA changes (Fig. 3b) in the experimental group in the early visual clusters detected in the whole-brain analysis (grey matter volume change cluster size: 1760 voxels; peak MNI = −11–69 12; FA change cluster size: 212 voxels; peak MNI = −12–75 1 see Supplementary Tables S1 and S4). Note that this was done only to assess between-subject consistency of observed effects and no statistical conclusions can be inferred from this particular analysis, since this particular ROI selection was not fully orthogonal to the data presented^96^. The obtained individual values were also correlated with each other, as well as with subjects’ age and behavioural variables - training-related changes (performance at baseline session subtracted from the performance in the after-training session) in tactile Braille word reading speed, tactile Braille letter reading speed and, as a control analyses, visual Braille word reading speed and visual Braille word reading accuracy (Supplementary Figure S2). To test for correlation between anatomical and behavioural changes at the whole-brain level, above-mentioned behavioural variables were also included in regression models, with training-related changes in grey matter volume or FA as dependent variables (scans from the baseline session subtracted from the scans in the after-training session; the same statistical thresholds as in other whole-brain analyses were used – see above).

Based on the results of the whole-brain analyses and our apriori expectation to find training-induced changes in the visual cortex, a region-of-interest (ROI) analysis (Figs 2c and 3d) was then conducted to describe the time-course of anatomical changes in the early visual cortex and to test whether this reorganization is specific to the experimental group. To avoid double-dipping^96,97^, the ROIs were defined based on the after-training vs. baseline contrast in 10 Braille learners who did not participate in the follow-up session. Then, only the remaining 19 subjects from the experimental group and the participants in the longitudinal control group were tested. Thus, definition of ROIs and further analysis were performed on independent datasets. ROIs were defined as 10 mm × 10 mm cubes centred on peaks of increase in grey matter (for VBM) or FA (for DTI) in the left hemisphere, following the training. In the case of DTI, the ROI was further constrained by including only voxels in which FA > 0.2 (i.e., excluding voxels with no data – see Data Preprocessing).

In the resting-state fMRI functional connectivity analysis, we compared the functional connectivity of two seeds: the peripheral and the central part of the early visual cortex. The analysis was performed on two datasets: before-training data from the experimental group and data from the control group of 31 subjects that were not familiar with Braille reading, neither in tactile nor in visual modality (see Subjects section). Seed definitions were based on the early visual cortex mask (V1 and V2 masks combined), as implemented in the Anatomy SPM toolbox^98^. In line with the principle of retinotopic organization of the early visual cortex^35^, the peripheral seed was defined as the anterior part of the mask (i.e., all voxels anterior to y = −85 MNI coordinate were included). The central seed was defined as the remaining, posterior part of the same mask (all voxels posterior to y = −85 MNI coordinate). The functional connectivity patterns of these two seeds were then compared with a parametric paired t-test. The comparison was assigned a false discovery rate threshold of q < 0.05 and an additional cluster-extent threshold of p < 0.05. Importantly, defining peripheral and central seeds based on different MNI coordinates (e.g., boundary at y = −80 or y = −90) did not significantly change the outcome.

All key comparisons in our study were performed in a repeated-measure design, in which each subject is his/her own control. Thus, the between-subject variability in demographic measures or behavioural outcomes of the Braille training did not affect findings reported in the study.

### Data availability

All data generated and analysed during the study are available from the corresponding author on request.

## Electronic supplementary material

Supplementary Information

## References

[1] Lövdén M, Bäckman L, Lindenberger U, Schaefer S, Schmiedek F (2010). A theoretical framework for the study of adult cognitive plasticity. Psychol. Bull..

[2] Zatorre RJ, Fields RD, Johansen-Berg H (2012). Plasticity in gray and white: neuroimaging changes in brain structure during learning. Nat. Neurosci..

[3] Kwok V (2011). Learning new color names produces rapid increase in gray matter in the intact adult human cortex. Proc. Natl. Acad. Sci. USA.

[4] Draganski B (2004). Neuroplasticity: changes in grey matter induced by training. Nature.

[5] Scholz J, Klein MC, Behrens TEJ, Johansen-Berg H (2009). Training induces changes in white-matter architecture. Nat. Neurosci..

[6] Hyde KL (2009). Musical Training Shapes Structural Brain Development. J. Neurosci..

[7] Heimler B, Striem-Amit E, Amedi A (2015). Origins of task-specific sensory-independent organization in the visual and auditory brain: neuroscience evidence, open questions and clinical implications. Curr. Opin. Neurobiol..

[8] Pascual-Leone A, Amedi A, Fregni F, Merabet LB (2005). The plastic human brain cortex. Annu. Rev. Neurosci..

[9] Sadato N (1996). Activation of the primary visual cortex by Braille reading in blind subjects. Nature.

[10] Büchel C, Price C, Friston K (1998). A multimodal language region in the ventral visual pathway. Nature.

[11] Poirier C (2006). Auditory motion perception activates visual motion areas in early blind subjects. Neuroimage.

[12] Renier LA (2010). Preserved functional specialization for spatial processing in the middle occipital gyrus of the early blind. Neuron.

[13] Reich L, Szwed M, Cohen L, Amedi A (2011). A ventral visual stream reading center independent of visual experience. Curr. Biol..

[14] Levänen S, Jousmäki V, Hari R (1998). Vibration-induced auditory-cortex activation in a congenitally deaf adult. Curr. Biol..

[15] Finney EM, Fine I, Dobkins KR (2001). Visual stimuli activate auditory cortex in the deaf. Nat. Neurosci..

[16] Karns CM, Dow MW, Neville HJ (2012). Altered Cross-Modal Processing in the Primary Auditory Cortex of Congenitally Deaf Adults: A Visual-Somatosensory fMRI Study with a Double-Flash Illusion. J. Neurosci..

[17] Scott GD, Karns CM, Dow MW, Stevens C, Neville HJ (2014). Enhanced peripheral visual processing in congenitally deaf humans is supported by multiple brain regions, including primary auditory cortex. Front. Hum. Neurosci..

[18] Bola Ł (2017). Task-specific reorganization of the auditory cortex in deaf humans. Proc. Natl. Acad. Sci. USA.

[19] Haslinger B (2005). Transmodal sensorimotor networks during action observation in professional pianists. J. Cogn. Neurosci..

[20] Siuda-Krzywicka K (2016). Massive cortical reorganization in sighted Braille readers. Elife.

[21] Amedi A (2007). Shape conveyed by visual-to-auditory sensory substitution activates the lateral occipital complex. Nat. Neurosci..

[22] Merabet LB (2008). Rapid and reversible recruitment of early visual cortex for touch. PLoS One.

[23] Zangenehpour S, Zatorre RJ (2010). Crossmodal recruitment of primary visual cortex following brief exposure to bimodal audiovisual stimuli. Neuropsychologia.

[24] Shimony J (2006). Diffusion tensor imaging reveals white matter reorganization in early blind humans. Cerebral Cortex.

[25] Jiang J (2009). Thick Visual Cortex in the Early Blind. J. Neurosci..

[26] Park H-J (2009). Morphological alterations in the congenital blind based on the analysis of cortical thickness and surface area. Neuroimage.

[27] Voss P, Pike BG, Zatorre RJ (2014). Evidence for both compensatory plastic and disuse atrophy-related neuroanatomical changes in the blind. Brain.

[28] Emmorey K, Allen JS, Bruss J, Schenker N, Damasio H (2003). A morphometric analysis of auditory brain regions in congenitally deaf adults. Proc. Natl. Acad. Sci. USA.

[29] Penhune VB, Cismaru R, Dorsaint-Pierre R, Petitto LA, Zatorre RJ (2003). The morphometry of auditory cortex in the congenitally deaf measured using MRI. Neuroimage.

[30] Li W (2015). Grey matter connectivity within and between auditory, language and visual systems in prelingually deaf adolescents. Restor. Neurol. Neurosci..

[31] Shiell MM, Champoux F, Zatorre RJ (2016). The Right Hemisphere Planum Temporale Supports Enhanced Visual Motion Detection Ability in Deaf People: Evidence from Cortical Thickness. Neural Plast..

[32] Shiell MM, Zatorre RJ (2017). White matter structure in the right planum temporale region correlates with visual motion detection thresholds in deaf people. Hear. Res..

[33] Bola Ł (2016). Braille in the Sighted: Teaching Tactile Reading to Sighted Adults. PLoS One.

[34] Bola Ł (2017). Universal Visual Features Might Be Necessary for Fluent Reading. A Longitudinal Study of Visual Reading in Braille and Cyrillic Alphabets. Front. Psychol..

[35] Wandell BA, Dumoulin SO, Brewer AA (2007). Visual field maps in human cortex. Neuron.

[36] Lewis JW, Van Essen DC (2000). Corticocortical connections of visual, sensorimotor, and multimodal processing areas in the parietal lobe of the macaque monkey. J. Comp. Neurol..

[37] Mesulam M (1998). From sensation to cognition. Brain.

[38] Ghazanfar AA, Schroeder CE (2006). Is neocortex essentially multisensory?. Trends Cogn. Sci..

[39] Kayser C, Logothetis NK (2007). Do early sensory cortices integrate cross-modal information?. Brain Struct. Funct..

[40] Murray MM (2015). The multisensory function of the human primary visual cortex. Neuropsychologia.

[41] Falchier A, Clavagnier S, Barone P, Kennedy H (2002). Anatomical Evidence of Multimodal Integration in Primate Striate Cortex. J. Neurosci..

[42] Rockland KS, Ojima H (2003). Multisensory convergence in calcarine visual areas in macaque monkey. Int. J. Psychophysiol..

[43] Henschke JU, Noesselt T, Scheich H, Budinger E (2015). Possible anatomical pathways for short-latency multisensory integration processes in primary sensory cortices. Brain Struct. Funct..

[44] Song S (2005). Demyelination increases radial diffusivity in corpus callosum of mouse brain. Neuroimage.

[45] Song S (2002). Dysmyelination revealed through MRI as increased radial (but unchanged axial) diffusion of water. Neuroimage.

[46] Nair G (2005). Myelination and long diffusion times alter diffusion-tensor-imaging contrast in myelin-deficient shiverer mice. Neuroimage.

[47] Eckert MA (2008). A cross-modal system linking primary auditory and visual cortices: evidence from intrinsic fMRI connectivity analysis. Hum. Brain Mapp..

[48] Saygin ZM (2016). Connectivity precedes function in the development of the visual word form area. Nat. Neurosci..

[49] Cohen LG (1997). Functional relevance of cross-modal plasticity in blind humans. Nature.

[50] Amedi A, Raz N, Pianka P, Malach R, Zohary E (2003). Early ‘visual’ cortex activation correlates with superior verbal memory performance in the blind. Nat. Neurosci..

[51] Raz N, Amedi A, Zohary E (2005). V1 activation in congenitally blind humans is associated with episodic retrieval. Cereb. Cortex.

[52] Bedny M, Pascual-Leone A, Dodell-Feder D, Fedorenko E, Saxe R (2011). Language processing in the occipital cortex of congenitally blind adults. Proc. Natl. Acad. Sci. USA.

[53] Zangaladze A, Epstein CM, Grafton ST, Sathian K (1999). Involvement of visual cortex in tactile discrimination of orientation. Nature.

[54] Merabet L (2004). Feeling by Sight or Seeing by Touch?. Neuron.

[55] Saito DN, Okada T, Honda M, Yonekura Y, Sadato N (2006). Practice makes perfect: the neural substrates of tactile discrimination by Mah-Jong experts include the primary visual cortex. BMC Neurosci..

[56] Stilla R, Sathian K (2008). Selective visuo-haptic processing of shape and texture. Hum. Brain Mapp..

[57] Sathian K (2011). Dual pathways for haptic and visual perception of spatial and texture information. Neuroimage.

[58] Eck J, Kaas AL, Goebel R (2013). Crossmodal interactions of haptic and visual texture information in early sensory cortex. Neuroimage.

[59] Eck J (2016). The Effect of Task Instruction on Haptic Texture Processing: The Neural Underpinning of Roughness and Spatial Density Perception. Cereb. Cortex.

[60] Pascual-Leone A, Hamilton R (2001). The metamodal organization of the brain. Prog. Brain Res..

[61] Driscoll I (2009). Longitudinal pattern of regional brain volume change differentiates normal aging from MCI. Neurology.

[62] Hutton C, Draganski B, Ashburner J, Weiskopf N (2009). A comparison between voxel-based cortical thickness and voxel-based morphometry in normal aging. Neuroimage.

[63] Teipel SJ (2010). Longitudinal changes in fiber tract integrity in healthy aging and mild cognitive impairment: A DTI follow-up study. J. Alzheimer’s Dis..

[64] Sexton CE (2014). Accelerated changes in white matter microstructure during aging: a longitudinal diffusion tensor imaging study. J. Neurosci..

[65] Grill-Spector K, Malach R (2004). The human visual cortex. Annu. Rev. Neurosci..

[66] Dehaene S, Cohen L (2011). The unique role of the visual word form area in reading. Trends Cogn. Sci..

[67] Price CJ, Devlin JT (2011). The interactive account of ventral occipitotemporal contributions to reading. Trends Cogn. Sci..

[68] Boyke J, Driemeyer J, Gaser C, Büchel C, May A (2008). Training-induced brain structure changes in the elderly. J. Neurosci..

[69] Driemeyer J, Boyke J, Gaser C, Büchel C, May A (2008). Changes in gray matter induced by learning–revisited. PLoS One.

[70] Sampaio-Baptista C (2014). Gray matter volume is associated with rate of subsequent skill learning after a long term training intervention. Neuroimage.

[71] Stehberg J, Dang PT, Frostig RD (2014). Unimodal primary sensory cortices are directly connected by long-range horizontal projections in the rat sensory cortex. Front. Neuroanat..

[72] Meredith MA, Lomber SG (2016). Species-dependent role of crossmodal connectivity among the primary sensory cortices. Hear. Res..

[73] Gomez-Robles A, Hopkins WD, Schapiro SJ, Sherwood CC (2015). Relaxed genetic control of cortical organization in human brains compared with chimpanzees. Proc. Natl. Acad. Sci..

[74] Smith SM (2006). Tract-based spatial statistics: voxelwise analysis of multi-subject diffusion data. Neuroimage.

[75] Smith SM (2004). Advances in functional and structural MR image analysis and implementation as FSL. Neuroimage.

[76] Heiervang E, Behrens TEJ, Mackay CE, Robson MD, Johansen-Berg H (2006). Between session reproducibility and between subject variability of diffusion MR and tractography measures. Neuroimage.

[77] Jovicich J (2014). Multisite longitudinal reliability of tract-based spatial statistics in diffusion tensor imaging of healthy elderly subjects. Neuroimage.

[78] Maier-Hein, K. *et al*. Tractography-based connectomes are dominated by false-positive connections. *bioRxiv* (2016).

[79] Smith SM (2002). Fast robust automated brain extraction. Hum. Brain Mapp..

[80] Behrens TEJ (2003). Characterization and propagation of uncertainty in diffusion-weighted MR imaging. Magn. Reson. Med..

[81] Alexander A, Lee J, Lazar M, Field A (2007). Diffusion tensor imaging of the brain. Neurotherapeutics.

[82] Engvig A (2012). Memory training impacts short-term changes in aging white matter: a longitudinal diffusion tensor imaging study. Hum. Brain Mapp..

[83] Madhyastha T (2014). Longitudinal reliability of tract-based spatial statistics in diffusion tensor imaging. Hum. Brain Mapp..

[84] Chao-Gan Y, Yu-Feng Z (2010). DPARSF: A MATLAB Toolbox for ‘Pipeline’ Data Analysis of Resting-State fMRI. Front. Syst. Neurosci..

[85] Hallquist MN, Hwang K, Luna B (2013). The nuisance of nuisance regression: spectral misspecification in a common approach to resting-state fMRI preprocessing reintroduces noise and obscures functional connectivity. Neuroimage.

[86] Friston KJ, Williams S, Howard R, Frackowiak RS, Turner R (1996). Movement-related effects in fMRI time-series. Magn. Reson. Med..

[87] Satterthwaite TD (2013). An improved framework for confound regression and filtering for control of motion artifact in the preprocessing of resting-state functional connectivity data. Neuroimage.

[88] Yan C-G (2013). A comprehensive assessment of regional variation in the impact of head micromovements on functional connectomics. Neuroimage.

[89] Power JD, Barnes KA, Snyder AZ, Schlaggar BL, Petersen SE (2012). Spurious but systematic correlations in functional connectivity MRI networks arise from subject motion. Neuroimage.

[90] Fox MD, Zhang D, Snyder AZ, Raichle ME (2009). The global signal and observed anticorrelated resting state brain networks. J. Neurophysiol..

[91] Murphy K, Birn RM, Handwerker DA, Jones TB, Bandettini PA (2009). The impact of global signal regression on resting state correlations: are anti-correlated networks introduced?. Neuroimage.

[92] Weissenbacher A (2009). Correlations and anticorrelations in resting-state functional connectivity MRI: a quantitative comparison of preprocessing strategies. Neuroimage.

[93] Genovese CR, Lazar NA, Nichols T (2002). Thresholding of statistical maps in functional neuroimaging using the false discovery rate. Neuroimage.

[94] Winkler AM, Ridgway GR, Webster MA, Smith SM, Nichols TE (2014). Permutation inference for the general linear model. Neuroimage.

[95] Eklund A, Nichols TE, Knutsson H (2016). Cluster failure: Why fMRI inferences for spatial extent have inflated false-positive rates. Proc. Natl. Acad. Sci. USA.

[96] Kriegeskorte N, Simmons WK, Bellgowan PSF, Baker CI (2009). Circular analysis in systems neuroscience: the dangers of double dipping. Nat. Neurosci..

[97] Thomas C, Baker CI (2013). Teaching an adult brain new tricks: A critical review of evidence for training-dependent structural plasticity in humans. NeuroImage.

[98] Eickhoff SB (2005). A new SPM toolbox for combining probabilistic cytoarchitectonic maps and functional imaging data. Neuroimage.

