# Supplementary material for: Structural reorganization of the early visual cortex following Braille training in sighted adults
Source: Sci Rep. 2017 Dec 12;7:17448. doi: 10.1038/s41598-017-17738-8 (PMC5727097; doi:10.1038/s41598-017-17738-8)
Supplement: Supplementary file 1 — Supplementary Information [file 41598_2017_17738_MOESM1_ESM.pdf]

Supplementary Information for

Structural reorganization of the early visual cortex following Braille reading training in sighted adults

Łukasz Bola, Katarzyna Siuda-Krzywicka, Małgorzata Paplińska, Ewa Sumera, Maria Zimmermann, Katarzyna Jednoróg, Artur Marchewka, Marcin Szwed

Supplementary Discussion

Lack of structural reorganization in the somatosensory cortex and the ventral visual cortex

In a number of studies reorganization induced by the tactile training has been found mainly in the somatosensory cortex[1,2] or the frontal lobe[3]. However, these studies used relatively simple tactile tasks, such as two-choice discrimination of tactile stimuli, and short training periods. It may be the case that at the initial stage of tactile Braille training plasticity occurs mostly in the somatosensory cortex[4]. However, with prolonged training, followed by the introduction of complex stimuli in the form of entire Braille words, the structural plasticity might switch to the visual cortex. Further studies with multiple scanning sessions will be required to test this possibility.

Based on previous reports, one may also have expected to find structural reorganization in the Visual Word Form Area[5,6] – a ventral visual stream region that was shown to be critical for tactile Braille reading in blind and sighted subjects[7–11]. However, several studies have already reported that functional and structural reorganization during learning might be found in different cortical locations[12,13]. It might be the case, that what needs to be primarily established in the course of anatomical reorganization is the efficient connectivity between visual and somatosensory cortices. Such connectivity might then facilitate functional recruitment of the ventral visual cortex for Braille reading.

Grey matter volume and FA decreases

In our study, we also detected grey matter volume and FA decreases with time (see Figures 2a and 3a in the main text as well as Supplementary Tables S1 and S4). No interaction between the experimental group and the control group was observed. Most likely the observed decreases are likely to be associated with non-specific, physiological effects, such as ageing[14-17]. It is interesting, however, that in within-group comparisons observed effects seem to be more extensive in the experimental group than in the control group (Supplementary Tables S1 and S4). It might be the case that our subject samples were too small to detect decreases specifically linked to the Braille training in the whole-brain interaction analysis. Training-related decreases can be sometimes observed, e.g. during motor training in adults, and further studies are needed to explore this possibility and the role of grey matter volume and FA decreases in learning (see also: [18-19]).

Impact of visual Braille reading on observed results

After training, we detected a progress in subjects' visual Braille reading performance (see Behavioral Results and Supplementary Figure S1). However, we argue that the observed structural reorganization is linked to tactile training and not to its visual counterpart. First, when our subjects checked their Braille exercises visually, they did so using foveal, and not peripheral vision. In line with well-known principle of retinotopic organization of the early visual cortex[20], a large number of studies demonstrate that perceptual training in the central visual field induces plasticity in the posterior part of the early visual cortex, which represents the foveal and para-foveal visual field[21–25]. This is also the case for letters and letter-like shapes[26–30]. In our study, we detected structural reorganization only in the most anterior part of the early visual cortex, which represents the far peripheries of the visual field. We found no structural reorganization in the central visual field. Second, our functional connectivity analysis showed that even before the onset of training, the peripheral part of the visual cortex had preferential connectivity with the primary somatosensory cortex and the primary motor cortex. The fact that only this subpart of the early visual cortex underwent structural plasticity suggests that this process was triggered by tactile input. Third, our subjects were familiar with visually presented Braille before the start of the training. Therefore, it seems unlikely that additional, slight increase in their visual Braille reading performance caused such profound reorganization in the early visual cortex, as it was observed in our study.

Our subjects were familiar with visually presented Braille before the onset of study (see Behavioral Results). The rationale to include such subjects in a study was to facilitate progress in tactile Braille learning. We cannot exclude that prior familiarity with visual Braille modulated the extent of structural reorganization observed in our study. One may speculate that structural reorganization induced by learning Braille in both tactile and visual modality should extend into the posterior early visual cortex and high-level visual regions (see for example [30]). However, it is unlikely that familiarity with visual Braille significantly affected the main result presented here, namely structural reorganization of the peripheral early visual cortex (see discussion above and in the main text).

Cellular basis of effects observed with structural neuroimaging

The cellular basis of experience-dependent, structural plasticity in the human brain remains unclear. Non-human data indicates that learning induces changes in a large number of cellular components[31]. In the case of grey matter, these changes can be broadly categorized

into neuronal (e.g., neurogenesis, synaptogenesis, alterations in neuronal morphology) and extra-neuronal effects (increases in glial cell size and number, angiogenesis). In the case of white matter, learning can affect the number of axons, axon diameter, the packing density of fibers, axon branching, axon trajectories and myelination. Alterations in all of these components might contribute to the effects observed on macroscopic level with structural MRI. However, several studies that combined high-resolution MRI with immunohistochemistry suggest that learning-induced changes in the grey matter volume detected with MRI might be attributed to the remodeling of neuronal processes rather than to increase in size or number of neurons or astrocytes[32]; changes in FA measure, in turn, might be a result of alterations in myelination rather than generation of new axonal pathways[33]. In line with the latter result, in our study an increase in FA was mostly driven by decreased radial diffusivity (Fig. 3d), a measure that is known to be sensitive to myelination processes[34–36]. While it is important to remember that radial diffusivity might be also modulated by many other factors, such a pattern of results might suggest that white matter reorganization observed in our study was driven by increased myelination in the early visual cortex.

Reliability of observed effects

Reliability of effects found with longitudinal structural neuroimaging was recently debated[37–39]. In our study, we aimed at increasing reliability of our results in several ways. First, we used two independent structural imaging methods, namely voxel-based morphometry (Fig. 2) and diffusion tensor imaging (Fig. 3), and the obtained results are highly congruent (Fig. 3c). Second, in both analyses we used preprocessing flows that are optimized specifically for longitudinal analyses and increase their reliability. Third, robustness of our main result was further confirmed in independent ROI analyses (Fig. 2c and Fig. 3d). Fourth, a control group was included in our study, and the interaction analyses confirmed that the effects observed in the early visual cortex were specific to the experimental group (Fig. 2c and Fig. 3d). Therefore we argue that changes in the peripheral early visual cortex detected in our study can be considered as reliable.

Supplementary References

1. Guic, E., Carrasco, X., Rodríguez, E., Robles, I. & Merzenich, M. M. Plasticity in primary somatosensory cortex resulting from environmentally enriched stimulation and

sensory discrimination training. *Biol. Res.* **41,** 425–437 (2008).

2. Pleger, B. *et al.* Functional imaging of perceptual learning in human primary and secondary somatosensory cortex. *Neuron* **40,** 643–653 (2003).

3. Sathian, K., Deshpande, G. & Stilla, R. Neural changes with tactile learning reflect decision-level reweighting of perceptual readout. *J. Neurosci.* **33,** 5387–5398 (2013).

4. Debowska, W. *et al.* Functional and Structural Neuroplasticity Induced by Short-Term Tactile Training Based on Braille Reading. *Front. Neurosci.* **10,** 460 (2016).

5. Cohen, L. *et al.* The visual word form area: spatial and temporal characterization of an initial stage of reading in normal subjects and posterior split-brain patients. *Brain* **123,** 291–307 (2000).

6. Dehaene, S. & Cohen, L. The unique role of the visual word form area in reading. *Trends Cogn. Sci.* **15,** 254–262 (2011).

7. Siuda-Krzywicka, K. *et al.* Massive cortical reorganization in sighted Braille readers. *Elife* **5,** e10762 (2016).

8. Büchel, C., Price, C. & Friston, K. A multimodal language region in the ventral visual pathway. *Nature* **394,** 274–277 (1998).

9. Reich, L., Szwed, M., Cohen, L. & Amedi, A. A ventral visual stream reading center independent of visual experience. *Curr. Biol.* **21,** 363–368 (2011).

10. Sadato, N. *et al.* Activation of the primary visual cortex by Braille reading in blind subjects. *Nature* **380,** 526–528 (1996).

11. Merabet, L. B. *et al.* Rapid and reversible recruitment of early visual cortex for touch. *PLoS One* **3,** e3046 (2008).

12. Thomas, A. G. *et al.* Functional but not structural changes associated with learning: an exploration of longitudinal voxel-based morphometry (VBM). *Neuroimage* **48,** 117–125 (2009).

13. Schmidt-Wilcke, T., Rosengarth, K., Luerding, R., Bogdahn, U. & Greenlee, M. W. Distinct patterns of functional and structural neuroplasticity associated with learning

Morse code. *Neuroimage* **51,** 1234–1241 (2010).

14. Driscoll, I. *et al.* Longitudinal pattern of regional brain volume change differentiates normal aging from MCI. *Neurology* **72,** 1906–1913 (2009).
15. Hutton, C., Draganski, B., Ashburner, J. & Weiskopf, N. A comparison between voxel-based cortical thickness and voxel-based morphometry in normal aging. *Neuroimage* **48,** 371–380 (2009).
16. Teipel, S. J. *et al.* Longitudinal changes in fiber tract integrity in healthy aging and mild cognitive impairment: A DTI follow-up study. *J. Alzheimer's Dis.* **22,** 507–522 (2010).
17. Sexton, C. E. *et al.* Accelerated changes in white matter microstructure during aging: a longitudinal diffusion tensor imaging study. *J. Neurosci.* **34,** 15425–36 (2014).
18. Hänggi, J., Koeneke, S., Bezzola, L. & Jäncke, L. Structural neuroplasticity in the sensorimotor network of professional female ballet dancers. *Hum. Brain Mapp.* **31,** 1196–1206 (2010).
19. Taubert, M. *et al.* Dynamic properties of human brain structure: learning-related changes in cortical areas and associated fiber connections. *J. Neurosci.* **30,** 11670–7 (2010).
20. Wandell, B. A., Dumoulin, S. O. & Brewer, A. A. Visual field maps in human cortex. *Neuron* **56,** 366–383 (2007).
21. Ahissar, M. & Hochstein, S. Task difficulty and the specificity of perceptual learning. *Nature* **387,** 401–406 (1997).
22. Crist, R. E., Kapadia, M. K., Westheimer, G. & Gilbert, C. D. Perceptual learning of spatial localization: specificity for orientation, position, and context. *J. Neurophysiol.* **78,** 2889–2894 (1997).
23. Crist, R. E., Li, W. & Gilbert, C. D. Learning to see: experience and attention in primary visual cortex. *Nat. Neurosci.* **4,** 519–525 (2001).
24. Furmanski, C. S., Schluppeck, D. & Engel, S. A. Learning strengthens the response of primary visual cortex to simple patterns. *Curr. Biol.* **14,** 573–578 (2004).
25. Schoups, A. A., Vogels, R. & Orban, G. A. Human perceptual learning in identifying

the oblique orientation: retinotopy, orientation specificity and monocularity. *J. Physiol.* **483** , 797–810 (1995).

26. Chang, C. H. C. *et al.* Adaptation of the human visual system to the statistics of letters and line configurations. *Neuroimage* **120,** 428–440 (2015).

27. Dehaene, S. *et al.* How learning to read changes the cortical networks for vision and language. *Science* **330,** 1359–1364 (2010).

28. Sigman, M. *et al.* Top-down reorganization of activity in the visual pathway after learning a shape identification task. *Neuron* **46,** 823–835 (2005).

29. Szwed, M. *et al.* Specialization for written words over objects in the visual cortex. *Neuroimage* **56,** 330–344 (2011).

30. Szwed, M., Qiao, E., Jobert, A., Dehaene, S. & Cohen, L. Effects of Literacy in Early Visual and Occipitotemporal Areas of Chinese and French Readers. *J. Cogn. Neurosci.* **26,** 459-475 (2014).

31. Zatorre, R. J., Fields, R. D. & Johansen-Berg, H. Plasticity in gray and white: neuroimaging changes in brain structure during learning. *Nat. Neurosci.* **15,** 528–536 (2012).

32. Lerch, J. P. *et al.* Maze training in mice induces MRI-detectable brain shape changes specific to the type of learning. *Neuroimage* **54,** 2086–2095 (2011).

33. Blumenfeld-Katzir, T., Pasternak, O., Dagan, M. & Assaf, Y. Diffusion MRI of Structural Brain Plasticity Induced by a Learning and Memory Task. *PLoS One* **6,** e20678 (2011).

34. Song, S. *et al.* Dysmyelination revealed through MRI as increased radial (but unchanged axial) diffusion of water. *Neuroimage* **17**, 1429-1436 (2002).

35. Song, S. *et al.* Demyelination increases radial diffusivity in corpus callosum of mouse brain. *Neuroimage* **26**, 132-140 (2005).

36. Nair, G. *et al.* Myelination and long diffusion times alter diffusion-tensor-imaging contrast in myelin-deficient shiverer mice. *Neuroimage* **28**, 165-174 (2005).

37. Thomas, C. & Baker, C. I. Teaching an adult brain new tricks: A critical review of evidence for training-dependent structural plasticity in humans. *NeuroImage* **73,** 225–236 (2013).

38. Fields, R. D. Changes in brain structure during learning: Fact or artifact? Reply to Thomas and Baker. *Neuroimage* **73,** 260–264 (2013).

39. Draganski, B. & Kherif, F. In vivo assessment of use-dependent brain plasticity—Beyond the 'one trick pony' imaging strategy. *Neuroimage* **73,** 255–259 (2013).

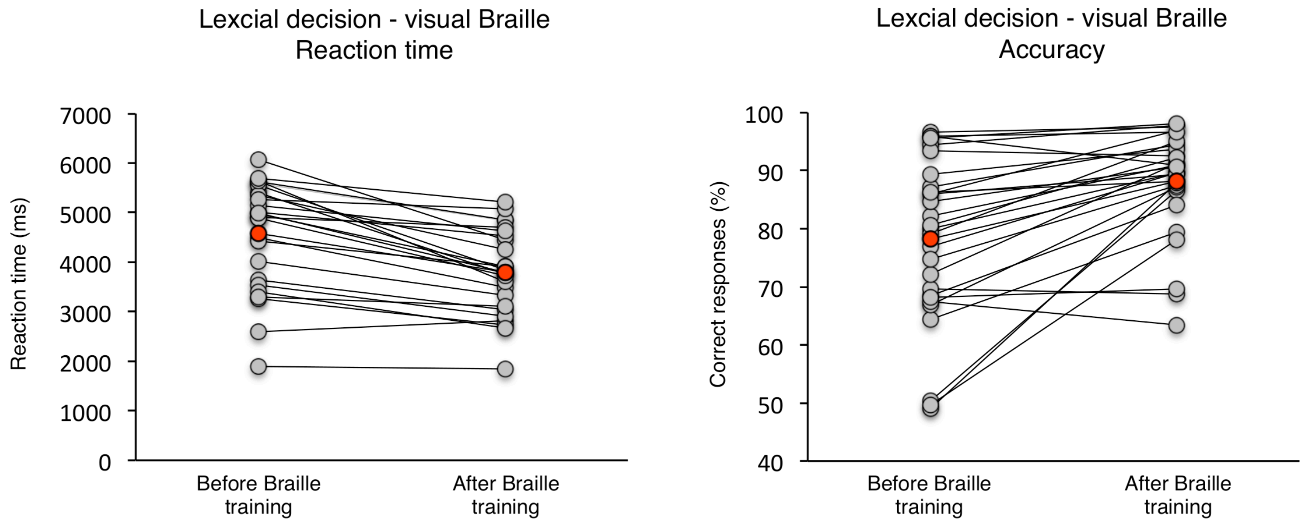

Supplementary Figure S1. Improvements in visual Braille reading skills, following the Braille training. Individual subjects' (a) reaction times and (b) accuracy in the lexical decision task in visual Braille are presented for baseline and after-training sessions. Group means are illustrated as red dots.

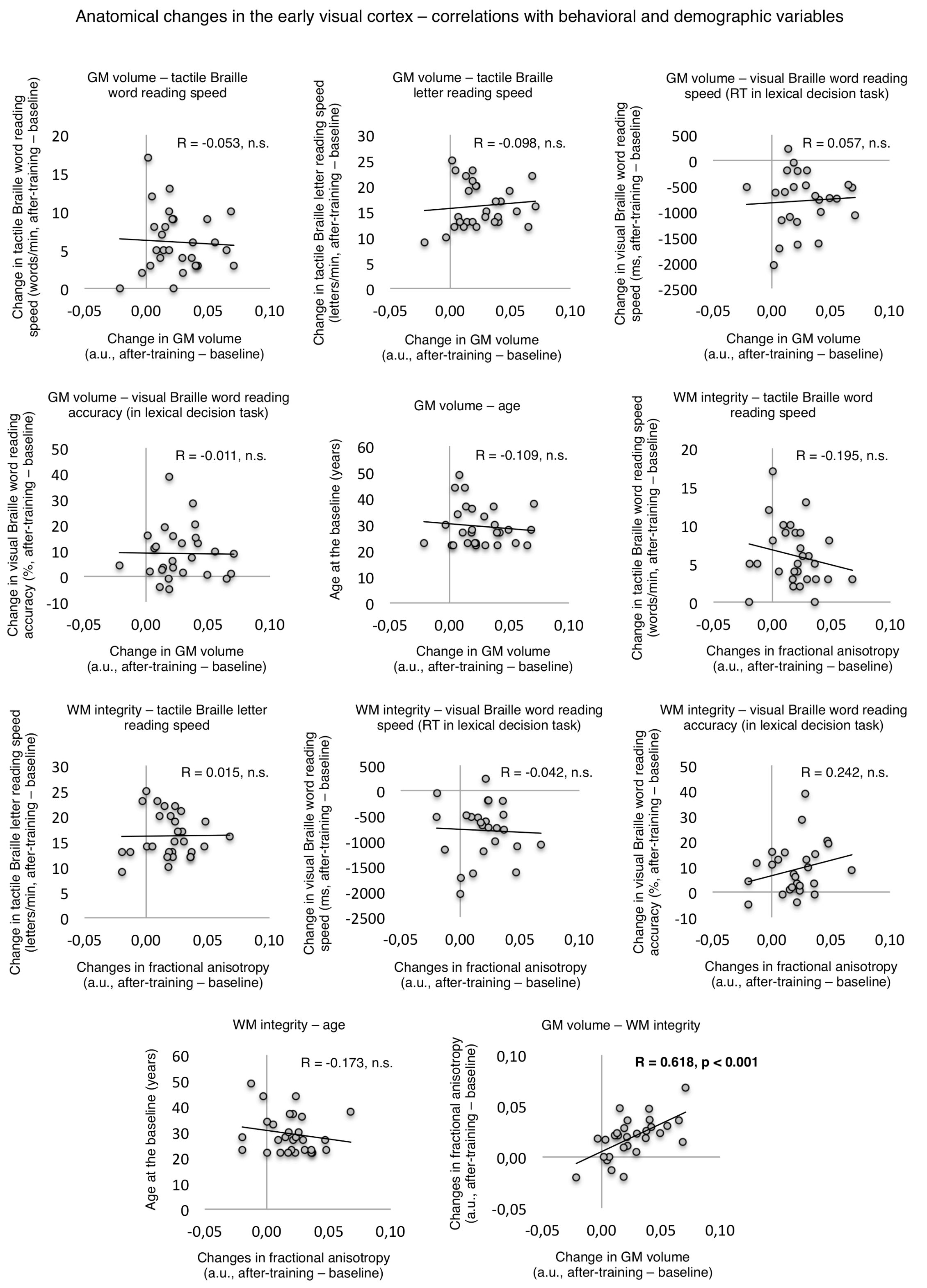

Supplementary Figure S2. Scatter plots for correlation analyses. Relationships between training-related anatomical reorganization in the peripheral early visual cortex, improvements in behavioral performance and age are depicted. The only significant result detected was correlation between grey matter volume change and fractional anisotropy change.

Supplementary Table S1. Grey matter volume changes in the experimental group, following the tactile Braille training (after-training vs. baseline), and in the control group (follow-up vs. baseline).

Contrast	Region	Brodmann's area	Extent	t-value	MNI Coordinates (x y z)		
Experimental group: after-training vs. baseline, increases							
	L Calcarine Gyrus	17	1760	6.56	-11	-69	12
	R Calcarine Gyrus	18		5.85	17	-71	17
	L Cerebelum (VIII)	NA	203	5.55	-33	-57	-44
	L Posterior-Medial Frontal	6	115	4.47	-9	2	57
Control group: follow-up vs. baseline, increases							
		No significant changes detected					
Experimental group: after-training vs. baseline, decreases							
	R Linual Gyrus	18	4286	7.91	20	-95	-11
	R Superior Occipital Gyrus	18		5.71	23	-95	14
	R Angular Gyrus	39		5.59	42	-59	36
	R Superior Occipital Gyrus	19		3.66	20	-86	42
	R Inferior Occipital Gyrus	19		3.07	39	-83	-2
	L Linual Gyrus	18	3341	6.48	-24	-95	-12
	L Superior Occipital Gyrus	18		4.61	-18	-93	26
	L Calcarine Gyrus	18		3.68	-6	-102	6
	L Middle Occipital Gyrus	19		3.35	-41	-80	3
	R Caudate Nucleus	NA	1497	6.47	21	18	2
	L Cerebelum (VII)	NA	6553	6.25	-6	-80	-36
	R Cerebelum (IX)	NA		5.83	8	-62	-47
	R Cerebelum (Crus 1)	NA		5.71	18	-77	-29
	NA	NA		3.67	-21	-69	-60
	NA	NA	781	6.20	8	14	26
	R ACC	NA		3.70	12	38	29
	NA	NA	2803	6.17	-20	20	0
	NA	NA		4.56	-42	-12	-15
	NA	48		4.24	-18	-8	23
	L Insula Lobe	44		3.78	-41	6	12
	NA	NA	558	5.67	-41	-59	-60
	NA	NA	429	5.03	-45	2	-48
	L Medial Temporal Pole	38		4.49	-53	11	-26
	NA	21		4.05	-68	-15	-20
	L Cerebelum (IV-V)	NA	388	4.98	-3	-57	-5
	R ParaHippocampal Gyrus	36	402	3.82	23	-36	-8
Control group: follow-up vs. baseline, decreases							
	Cerebellum	NA	30	8.57	39	-52	-59
	Anterior cingulate cortex	32	38	7.35	-8	39	-6
	Anterior cingulate cortex	32	34	6.89	10	38	-3

Threshold: false discovery rate of q<0.05, cluster extent threshold of p<0.05. Table shows all local maxima separated by more than 24 mm. Regions were labeled using the Anatomy SPM toolbox and Brodmann's classification. NA, not applicable.

Supplementary Table S2. Grey matter volume changes in the experimental group - follow-up vs. baseline (19 subjects included in the analysis).

Contrast	Region	Brodmann's area	Extent	t-value	MNI Coordinates (x y z)		
Experimental group: follow-up vs. baseline, increases							
	L Cerebelum (VIII)	NA	317	5.62	-35	-56	-42
	L Linual Gyrus	17	88	4.69	-2	-72	5
	R Calcarine Gyrus	17	83	4.59	18	-75	11
	R Rolandic Operculum	NA	61	4.54	56	-3	21
Experimental group: follow-up vs. baseline, decreases							
	R Cerebelum (IX)	NA	6836	7.48	9	-62	-45
	L Cerebelum (Crus 2)	NA		6.84	-6	-78	-30
	R Cerebelum (Crus 1)	NA		3.88	29	-78	-32
	NA	NA	3562	7.47	-12	23	26
	NA	NA		7.25	14	12	35
	R ACC	NA		5.58	17	38	14
	L ACC	NA		5.10	-14	44	5
	L MCC	6		5.02	-11	2	39
	R Superior Medial Gyrus	8		3.72	8	26	56
	R Superior Orbital Gyrus	10		3.68	18	59	2
	R Rectal Gyrus	11		2.97	11	32	-11
	R Superior Medial Gyrus	9		2.70	12	50	38
	L IFG (p. Opercularis)	NA	6075	7.23	-35	2	29
	NA	NA		6.45	-41	-24	-3
	NA	NA		5.59	-26	18	5
	L Superior Temporal Gyrus	NA		5.58	-47	-44	27
	L Superior Frontal Gyrus	10		4.57	-26	45	5
	L Middle Temporal Gyrus	NA		4.43	-42	-3	-23
	NA	NA		4.10	-23	-12	-36
	L Inferior Parietal Lobule	40		3.48	-48	-47	51
	L IFG (p. Opercularis)	44		3.46	-47	5	8
	R Inferior Occipital Gyrus	18	5188	7.16	27	-92	-8
	R Middle Occipital Gyrus	19		6.04	39	-74	18
	R Inferior Parietal Lobule	39		4.48	48	-50	44
	R Cuneus	18		3.83	8	-90	20
	R Middle Temporal Gyrus	37		3.49	56	-53	9
	NA	19	5596	6.85	-36	-66	21
	L Linual Gyrus	18		6.58	-18	-96	-14
	L Inferior Occipital Gyrus	19		5.65	-42	-78	-2
	L Superior Occipital Gyrus	18		4.88	-11	-96	11
	L Inferior Parietal Lobule	39		4.35	-39	-59	45
	L Inferior Temporal Gyrus	37		3.89	-59	-54	-8
	NA	NA	1635	6.42	-23	-14	51
	NA	7		5.49	-29	-45	47
	NA	NA		3.05	-39	-17	32

NA	NA	1980	6.15	29	18	-2
NA	NA		4.28	21	3	17
R Rolandic Operculum	6		3.74	47	2	14
NA	47	552	5.38	-53	35	-9
R Middle Frontal Gyrus	8	658	5.18	48	20	42
R Middle Frontal Gyrus	9		3.53	41	42	33
L Inferior Temporal Gyrus	37	994	5.08	-53	-53	-21
L Cerebelum (VIII)	NA		4.90	-44	-47	-47
L Inferior Temporal Gyrus	20		3.93	-47	-23	-29
R ParaHippocampal Gyrus	18	1187	4.88	20	-42	-2
R Precuneus	7	382	4.83	12	-60	48
L MCC	31	599	4.65	-2	-33	39
R Precuneus	23		3.81	9	-51	21
NA	21	1201	4.64	-65	-12	-27
L Medial Temporal Pole	38		4.32	-54	12	-27
L Superior Temporal Gyrus	41		2.94	-62	-5	2
L Angular Gyrus	7	484	4.44	-27	-57	39
L Superior Parietal Lobule	7		2.76	-15	-77	48

Threshold: false discovery rate of $q<0.05$, cluster extent threshold of $p<0.05$. Table shows all local maxima separated by more than 24 mm. Regions were labeled using the Anatomy SPM toolbox and Brodmann's classification. NA, not applicable.

Supplementary Table S3. Grey matter volume changes in the experimental group - follow-up vs. after-training (19 subjects included in the analysis).

Contrast	Region	Brodmann's area	Extent	t-value	MNI Coordinates (x y z)		
Experimental group: follow-up vs. after-training, increases							
	R Cerebelum (Crus 2)	NA	57	5.51	39	-80	-47
Experimental group: follow-up vs. after-training, decreases							
	L IFG (p. Opercularis)	NA	209	6.93	-38	0	29
	NA	NA	84	5.67	-12	17	29
	NA	8	87	5.23	-20	30	32
	NA	NA	95	5.22	-23	-14	51

Threshold: false discovery rate of q<0.05, cluster extent threshold of p<0.05. Table shows all local maxima separated by more than 24 mm. Regions were labeled using the Anatomy SPM toolbox and Brodmann's classification. NA, not applicable.

Supplementary Table S4. Fractional anisotropy changes in the experimental group, following the tactile Braille training (after-training vs. baseline), and in the control group (follow-up vs. baseline).

Contrast	Hemisphere	Extent	t-value	MNI coordinates (x y z)		
Experimental group: after-training vs. baseline , increases						
	Left	212	5.04	-12	-75	1
	Left	208	4.6	-21	13	14
	Right	172	4.77	15	-25	11
	Right	119	5.44	34	0	3
	Right	90	4.71	30	-57	25
	Right	72	4.58	35	-4	22
	Right	68	5.38	36	-54	33
Control group: follow-up vs. baseline , increases						
	No significant changes detected					
Experimental group: after-training vs. baseline , decreases						
	Right	283	5.04	18	9	42
	Left	114	4.48	-15	-30	29
	Left	74	5.43	-21	-18	-5
Control group: follow-up vs. baseline , decreases						
	No significant changes detected					

Threshold: $p < 0.001$ voxelwise, cluster extent threshold of $p<0.05$. Table shows all local maxima separated by more than 24 mm.

Supplementary Table S5. Fractional anisotropy changes in the experimental group - follow-up vs. baseline (19 subjects included in the analysis).

Contrast	Hemisphere	Extent	t-value	MNI coordinates (x y z)		
Experimental group: follow-up vs. baseline, increases						
	Left	550	6.81	-11	-60	-28
	Left	295	5.72	-20	9	14
	Right	279	5.79	20	-30	10
	Left	215	5.75	-13	-72	3
	Left	98	5.27	-37	-26	33
	Right	97	5.47	54	7	28
	Left	90	5.1	-13	-59	30
	Left	79	5.36	-23	-33	2
	Left	77	8.14	-51	-54	4
Experimental group: follow-up vs. baseline, decreases						
	Right	343	4.95	18	7	42
	Left	150	4.59	-26	-19	23
	Right	125	4.38	26	-26	39
	Left	122	5.11	-16	-37	29

Threshold: $p < 0.001$ voxelwise, cluster extent threshold of $p<0.05$. Table shows all local maxima separated by more than 24 mm.

Supplementary Table S6. Fractional anisotropy changes in the experimental group - follow-up vs. after-training (19 subjects included in the analysis).

Contrast	Hemisphere	Extent	t-value	MNI coordinates (x y z)
Experimental group: follow-up vs. baseline, increases				
	No significant changes detected			
Experimental group: follow-up vs. baseline, decreases				
	No significant changes detected			

Threshold: $p < 0.001$ voxelwise, cluster extent threshold of $p<0.05$. Table shows all local maxima separated by more than 24 mm.

Supplementary Table S7. Differences in functional connectivity of the anterior part of the early visual cortex (peripheral visual field) and the posterior part of the early visual cortex (central visual field) in the experimental group, before the onset of Braille training.

Contrast	Region	Brodmann's area	Extent	t-value	MNI Coordinates (x y z)		
Experimental group, the early visual cortex: peripheral > central							
	R Calcarine Gyrus	17	10399	17.65	15	-69	12
	L Linual Gyrus	19		16.13	-15	-66	-3
	R Fusiform Gyrus	19		9.87	24	-45	-6
	R Precuneus	NA		9.59	15	-54	54
	L Cuneus	19		9.47	-12	-78	30
	L Fusiform Gyrus	37		8.51	-30	-48	-12
	NA	7		8.26	-15	-54	54
	R Superior Frontal Gyrus	6		6.67	18	-3	66
	L Superior Frontal Gyrus	NA		6.33	-18	-3	60
	NA	NA		6.14	18	-33	42
	NA	NA		6.09	-21	-18	-3
	L Insula Lobe	13		6.08	-36	12	9
	R MCC	8		6.05	6	12	39
	L Cerebelum (VII)	NA		5.34	-42	-45	-36
	L Precentral Gyrus	NA		5.21	-60	0	39
	NA	NA		5.15	-15	-15	39
	R Middle Temporal Gyrus	19		5.07	42	-72	21
	L Middle Occipital Gyrus	19		5.05	-39	-75	15
	L Postcentral Gyrus	40		4.94	-63	-24	30
	R Hippocampus	NA		4.77	27	-21	-9
	R Cerebelum (Crus 2)	NA		4.71	42	-42	-33
	L Rolandic Operculum	4		4.69	-42	-12	18
	R Middle Frontal Gyrus	NA		4.53	51	-6	57
	L Postcentral Gyrus	NA		4.52	-45	-24	66
	R Thalamus	NA		4.47	9	-15	24
	R Postcentral Gyrus	1		4.25	30	-42	69
	R PCC	23		3.93	9	-42	21
	L Middle Temporal Gyrus	22		3.72	-57	-39	12
	NA	NA		3.56	36	-12	39
	R Inferior Temporal Gyrus	37		3.46	51	-51	-6
	NA	NA		3.40	9	-66	-24
	NA	NA		3.08	-30	-45	30
	NA	NA		2.96	-33	0	-18
	R Insula Lobe	6	1145	5.85	48	-3	6
	R Rolandic Operculum	40		5.64	57	-24	24
	R Postcentral Gyrus	NA		3.92	63	-24	48
Experimental group, the early visual cortex: peripheral < central							
	NA	NA	6517	17.97	12	-105	-3
	L Middle Occipital Gyrus	18		16.65	-15	-99	9

R Inferior Occipital Gyrus	18		14.92	39	-93	-3
NA	18		11.42	-18	-99	-15
L Cerebelum (Crus 2)	NA		11.03	-39	-81	-42
R Cerebelum (Crus 2)	NA		9.55	12	-84	-30
R Cerebelum (Crus 1)	NA		9.20	36	-81	-30
R Cuneus	18		9.15	21	-93	18
R Angular Gyrus	39		7.92	51	-63	45
L Angular Gyrus	NA		7.74	-54	-69	33
R Middle Temporal Gyrus	21		7.06	69	-27	-12
L Cerebelum (Crus 1)	NA		6.10	-18	-78	-27
R Inferior Temporal Gyrus	NA		5.84	57	-69	-12
NA	NA		5.83	-6	-84	-48
L Superior Parietal Lobule	NA		5.71	-30	-72	57
R Superior Occipital Gyrus	NA		5.34	33	-81	48
NA	37		5.26	-57	-63	-21
NA	NA		5.17	-54	-78	0
NA	NA		5.05	45	-18	-15
R Middle Temporal Gyrus	NA		4.90	69	-48	6
NA	NA		3.99	48	-6	-48
R SupraMarginal Gyrus	NA		3.64	66	-45	36
L Superior Temporal Gyrus	NA		3.38	-66	-51	18
L SupraMarginal Gyrus	39		2.90	-45	-45	33
R Superior Medial Gyrus	9	3998	7.25	9	57	33
NA	10		7.09	-30	63	6
L Posterior-Medial Frontal	8		6.81	-9	21	57
NA	NA		6.39	33	63	6
R Middle Frontal Gyrus	8		6.31	45	15	45
L Superior Frontal Gyrus	9		6.24	-18	51	33
NA	10		6.08	-48	51	-9
NA	47		5.73	-48	24	-15
L Middle Frontal Gyrus	6		5.27	-42	12	48
R Middle Frontal Gyrus	NA		4.77	54	33	27
NA	11		4.64	-18	60	-18
NA	47		4.34	33	27	-21
L IFG (p. Triangularis)	44		4.18	-45	18	12
R Superior Frontal Gyrus	8		3.97	21	33	54
NA	10		3.81	9	69	-9
NA	NA		3.07	-18	30	-27
R IFG (p. Triangularis)	44		3.01	60	21	6
L Middle Temporal Gyrus	21	885	5.65	-63	-39	-6
NA	20		5.16	-51	-15	-39
NA	NA		3.83	-27	-18	-42

Threshold: false discovery rate of q<0.05, cluster extent threshold of p<0.05. Table shows all local maxima separated by more than 24 mm. Regions were labeled using the Anatomy SPM toolbox and Brodmann's classification. NA, not applicable.

Supplementary Table S8. Differences in functional connectivity of the anterior part of the early visual cortex (peripheral visual field) and the posterior part of the early visual cortex (central visual field) in the control group.

Contrast	Region	Brodmann's area	Extent	t-value	MNI Coordinates (x y z)		
Control group, the early visual cortex: peripheral > central							
	R Calcarine Gyrus	18	14959	18.04	15	-75	21
	L Fusiform Gyrus	19		16.43	-18	-51	-6
	L Calcarine Gyrus	17		14.55	-15	-72	12
	R Linual Gyrus	19		14.02	15	-66	-3
	R Cerebelum (IV-V)	NA		11.47	12	-42	-6
	R Insula Lobe	13		7.85	42	-9	6
	L Insula Lobe	13		7.67	-33	9	12
	R MCC	31		7.49	9	-42	45
	R MCC	6		7.45	9	9	42
	L Rolandic Operculum	NA		7.20	-42	-21	21
	L SupraMarginal Gyrus	40		6.82	-63	-30	30
	L Superior Temporal Gyrus	6		6.58	-54	-3	6
	NA	31		6.29	-15	-36	42
	R Postcentral Gyrus	NA		6.28	24	-45	75
	R Rolandic Operculum	40		6.03	51	-27	27
	L Posterior-Medial Frontal	NA		5.88	-12	0	54
	L Superior Parietal Lobule	7		5.87	-27	-51	69
	L Middle Occipital Gyrus	19		5.79	-39	-75	15
	NA	NA		5.77	42	-54	0
	L Fusiform Gyrus	36		5.31	-30	-6	-33
	R Cerebelum (VII)	NA		5.25	42	-45	-42
	R Rolandic Operculum	1		5.19	69	-12	18
	R Superior Frontal Gyrus	6		5.17	18	0	63
	R Precentral Gyrus	NA		5.06	48	-18	63
	L Precentral Gyrus	6		5.05	-42	-15	66
	R ACC	32		4.91	6	33	21
	R Medial Temporal Pole	36		4.74	27	0	-33
	L Cerebelum (VIII)	NA		4.53	-42	-48	-42
	L Hippocampus	NA		4.47	-24	-24	-9
	L Superior Frontal Gyrus	NA		4.41	-15	-15	78
	R Rectal Gyrus	48		4.33	12	15	-9
	L Precentral Gyrus	6		4.29	-60	3	36
	R Hippocampus	54		3.89	27	-21	-12
	R Postcentral Gyrus	NA		3.75	63	-12	45
	R Middle Occipital Gyrus	19		3.67	39	-75	27
	R IFG (p. Opercularis)	6		3.44	63	9	33
	Cerebellar Vermis (8)	NA		3.39	0	-69	-24
	NA	NA		2.93	-30	-45	18

Control group, the early visual cortex: peripheral < central

L Middle Occipital Gyrus	18	4290	12.22	-18	-96	3
NA	NA		11.29	9	-99	-6
R Middle Occipital Gyrus	18		11.09	33	-96	6
R Cerebelum (Crus 1)	NA		9.96	42	-75	-33
L Cerebelum (Crus 2)	NA		9.50	-18	-84	-33
R Cerebelum (Crus 2)	NA		9.00	12	-87	-27
NA	NA		6.91	-39	-87	-15
L Cerebelum (Crus 2)	NA		6.10	-51	-66	-42
R Inferior Temporal Gyrus	21		5.62	66	-33	-18
R Inferior Temporal Gyrus	20		5.51	54	-15	-30
NA	NA		4.71	36	-18	-48
R Cuneus	NA		4.65	12	-99	24
NA	NA		3.99	-33	-81	-54
NA	NA		3.77	18	-81	-54
L Inferior Temporal Gyrus	37		3.54	-57	-66	-15
NA	NA		3.53	42	-69	-57
R Inferior Temporal Gyrus	NA		3.36	63	-60	-12
NA	10	3131	9.84	-45	54	0
NA	10		7.96	12	69	-9
NA	NA		7.74	48	54	-9
NA	NA		6.97	33	66	12
NA	11		6.58	-18	57	-18
NA	NA		6.20	-18	66	24
L Superior Medial Gyrus	NA		5.77	-6	48	54
L Middle Frontal Gyrus	8		5.11	-39	21	45
L Superior Medial Gyrus	NA		4.86	-3	27	66
NA	45		4.06	-57	18	3
R Superior Medial Gyrus	8		3.76	12	36	42
NA	21	475	4.79	-66	-30	-18
NA	NA		4.39	-60	-12	-33
NA	NA		3.43	-36	-24	-33
L Angular Gyrus	NA	181	4.30	-51	-69	42

Threshold: false discovery rate of q<0.05, cluster extent threshold of p<0.05. Table shows all local maxima separated by more than 24 mm. Regions were labeled using the Anatomy SPM toolbox and Brodmann's classification. NA, not applicable.
